# Natural genetic variation of a single amino acid in beet necrotic yellow vein virus P31 protein modulates evasion of plant ubiquitination-mediated antiviral immunity

**DOI:** 10.1371/journal.ppat.1013840

**Published:** 2026-01-02

**Authors:** Zhi-Hong Guo, Xin-Yu Qin, Meng-Ke Zhou, Xiu-Qi Zhang, Zhi-Yao Li, Chuan Zheng, Zong-Ying Zhang, Qian Chen, Xian-Bing Wang, Cheng-Gui Han, Ying Wang

**Affiliations:** 1 State Key Laboratory of Agricultural and Forestry Biosecurity, College of Plant Protection, China Agricultural University, Beijing, China; 2 State Key Laboratory of Plant Environmental Resilience, College of Biological Sciences, China Agricultural University, Beijing, China; University of Kentucky, UNITED STATES OF AMERICA

## Abstract

Plant virus evolution frequently leads to the emergence of highly virulent strains, posing persistent threats to global crop production. However, the mechanisms by which viral evolution enables evasion of plant defense responses remain poorly understood. Here, we found that *Benyvirus necrobetae* (beet necrotic yellow vein virus, BNYVV), causing the most destructive rhizomania disease in sugar beet worldwide, exhibits distinct pathogenicity associated with different virus isolates. Two Chinese isolates from Inner Mongolia (NM) and Xin Jiang (XJ) exhibit significantly higher virulence than the Japanese isolate O11 and Germany isolate OW1. Moreover, the high pathogenicity is attributed to the Arg-147 residue in P31^NM^ and P31^XJ^. The P31 proteins of these isolates exhibit enhanced protein stability, compared with P31^O11^ and P31^OW1^ carrying a Lys-147 residue. Furthermore, the E3 ubiquitin ligase Hmg-CoA reductase degradation 1 (HRD1) targets the P31^O11^ Lys-147 for ubiquitination and subsequent degradation. Whereas, the P31^NM^ Arg-147 evades HRD1-mediated degradation, conferring a virulence advantage. Overexpression of *HRD1* obviously inhibits BNYVV^O11^ infection, rather than BNYVV^NM^. Moreover, knockout of *NbHRD1* in *Nicotiana benthamiana* enhanced susceptibility to BNYVV infection. Collectively, our findings demonstrate an ongoing evolutionary arms race, in which HRD1 is a critical antiviral component by ubiquitinating viral proteins, while natural mutations in viral genomes enable evasion of host defense.

## Author summary

Natural variation of plant viruses usually produces newly emerging hyper-virulent strains triggering devastating losses in crop production worldwide. However, how virus variation affects the arm race of viruses and plant immunity remains largely unknown. Here, our study mainly focused on variation of *Benyvirus necrobetae* (beet necrotic yellow vein virus, BNYVV), and uncovered a critical molecular arms race between point mutation of P31, a pathogenicity factor of BNYVV, and a host E3 ligase, Hmg-CoA reductase degradation 1 (HRD1). Chinese isolates from Inner Mongolia and Xin Jiang demonstrated dramatically higher virulence compared to isolates from Japanese and Germany. The reason lies in a single amino acid variation leading to unprecedented stability and pathogenicity, which enable BNYVV to evade the plant defense system. We further demonstrate that *HRD1* as an E3 ligase targets and degrades the Japanese isolate, but the Inner Mongolia isolate evades this degradation. Overexpression of *HRD1* effectively blocks infection by the Japanese isolate but fails to inhibit the Inner Mongolia isolate, while knocking out *NbHRD1* plants became susceptible in BNYVV infection. Our finding reveals an intense, ongoing evolutionary battle at the molecular level, highlighting a rapidly shifting threat to global food security.

## Introduction

Plant viruses cause significant economic losses by reducing crop yield and quality worldwide [1]. RNA viruses exhibit high genetic variability due to lack of proofreading activity of RNA-dependent RNA polymerases (RdRp) [2]. Accumulation of genetic viral mutations during infections greatly affects virus virulence, transmission, host range and interaction with the host defense systems [3,4]. For instance, variations in the open reading frame 5 (ORF 5) of *Polerovirus TUYV* (turnip yellows virus) leads to new host adaptation and improved aphid transmission [5]. Moreover, increasing evidence demonstrates that highly virulent RNA virus strains usually break host resistance and cause virus disease epidemic [6,7]. The viral genome-linked protein (VPg) of *Sobemovirus RYMV* (rice yellow mottle virus) plays a key role in hypervirulent pathotype and the 49^th^ residue of VPg is responsible for breaking the eIF(iso)4G-mediated *Rymv1–2* resistance [8,9]. The high genetic divergence in RNA2 of *Fabavirus betaviciae* (broad bean wilt virus 2) determines pathogenic diversification among the virus isolates [10]. However, despite extensive analyses about virus genetic diversity, it remains largely underdefined on how random viral virulence mutations evade host defense system. Thus, exploring the mechanism would provide deep understanding of severe disease epidemic caused by plant viruses and their virulent strains worldwide.

Sugar beet (*Beta vulgaris*, *B. vulgaris*) is an economically important crop that provides approximately 35% of the annual sugar consumption in the world [11,12]. However, rhizomania caused by *Benyvirus necrobetae* (beet necrotic yellow vein virus, BNYVV) is a globally important sugar beet disease transmitted by the plasmodiophorid protist *Polymyxa betae* Keskin (*P. betae*) [13]. *B. necrobetae* is the type member of genus *Benyvirus* in family *Benyviridae*, and its genome is comprised of four to five single-stranded genomic RNAs [14]. RNA1 and 2 encoded house-keeping components for viral RNA replication, virion assembly, and cell-to-cell movement [15]. RNA3, 4, and 5 are non-essential components for virus infection, but play crucial roles in virus transmission and pathogenicity [16]. RNA3-encoded P25 is the virulence factor of BNYVV and it is responsible for rhizomania symptoms. RNA4-encoded P31 greatly increases the transmission rate by *P. betae* [17]. Previous studies showed that genetic diversity and geographical distribution of BNYVV is complicated [17,18]. BNYVV isolates are classified into four genotypes (A-, B-, P-, and J-type) based on the minor changes in the CP sequence and the presence of the RNA5 species [17,18]. Among the four genotypes of BNYVV, the A type strains distribute worldwide [19], while the B type strains are only found in China, Japan, and Europe [20,21]. The P and J type strains harboring RNA5 are widely distributed in Asia [18,22], but are present in small areas of France [19], Germany [20], and the United Kingdom [23]. In addition, both P25 and P31 are subjected to varied selection pressures [18]. Although many BNYVV isolates have been generated, the involvement of genetic variation in viral pathogenesis and host defense remains largely unknown.

The P31 of BNYVV mainly mediates efficient vector transmission, symptom severity and silencing suppression in roots [24]. Based on amino acids (aa) sequences, the P31 proteins from 74 BNYVV isolates are divided into four groups (I, II, III, and IV) [17,18]. Until now, only P31^O11^ of RNA4 from the Japanese O11 isolate, a member of group II, has been extensively studied in viral pathogenesis [24,25]. For instance, the RNA4^O11^ isolate induces severe symptoms such as stunting and curling in infected *Nicotiana benthamiana* (*N*. *benthamiana*) plants [24]. Moreover, the presence of P31^O11^ up-regulates the expression of host defense gene *pathogenesis-related protein 10* [26]. In addition, RNA transcriptomic analyses reveal that genes related to ubiquitination and 26S proteasome system (UPS) are significantly increased by BNYVV containing RNA4^O11^ isolate [27]. These results indicate that P31 is also involved in viral pathogenesis and might be a main target of host defense.

The ubiquitin (Ub)-UPS-mediated degradation is a highly conserved pathway for fine-tuning protein abundance in eukaryotic cells [28,29]. Substrate proteins are ubiquitinated through three enzyme complexes and then recognized and degraded through the 26S proteasome complex [30]. Increasing evidence demonstrates that ubiquitination-mediated degradation plays critical roles in the interactions between virus and host antiviral immunity [31–33]. For instance, the *N. benthamiana* ubiquitin E3 ligase containing RING domain 1 (NbUbE3R1) mediates degradation of the RNA replication protein of *Potexvirus bambusae* (bamboo mosaic virus) to inhibit virus infection [34]. Arabidopsis RING finger E3 ligase RKP inhibits *Curtovirus betae* (beet severe curly top virus) infection by triggering viral encoded C4 protein degradation [35]. Our previous studies reveal that the movement proteins of BNYVV and *Potexvirus ecspotati* (potato virus X) are degraded through the 26S proteasome [36]. To counter defense against host degradation, some plant viruses-encoded proteins interfere with enzyme activities of host E3 ligases or competitively bind to the E3 ligase substrate recognition site [33,37,38]. However, how plant viruses and their natural variation evade host antiviral ubiquitin/proteasome defense mechanism remains largely unknown.

Here, we compared the virulence of BNYVV RNA4 isolates belonging group I and group II, including two Chinese isolates from Inner Mongolia (RNA4^NM^) and Xin Jiang (RNA4^XJ^), as well as a Japanese isolate O11 (RNA4^O11^) and a Germany isolate OW1 (RNA4^OW1^). Our results revealed that the RNA4^NM^ and RNA4^XJ^ isolates caused severe symptoms due to abundant P31 accumulation. We further found that the E3 ligase Hmg-CoA reductase degradation 1 (HRD1), a core component of the endoplasmic reticulum-associated degradation (ERAD) pathway, interacted with P31 in the ER. HRD1 recognized P31^O11^ Lys-147 for ubiquitination and protein degradation. The P31^NM^ with Arg-147 was resistant to HRD1-triggered ubiquitination and protein degradation. Our study demonstrates that natural variation at codon 147 of BNYVV P31 determines the arm race with host antiviral immunity.

## Results

### Genetic variation of BNYVV RNA4 determines virus virulence

**Fig 1:**
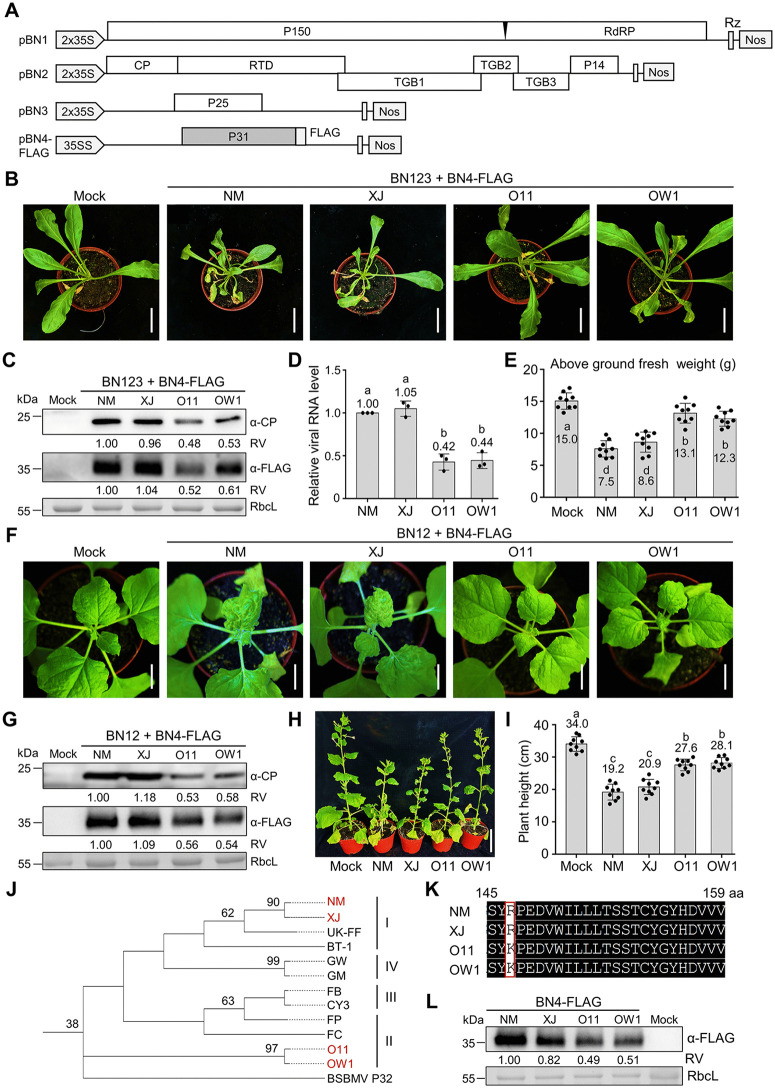
BNYVV RNA4 isolates in different regions cause distinct virus infectivity. **(A) **Schematic representation of the pBN1, pBN2, pBN3, and pBN4-FLAG constructs used to express BNYVV RNA1, RNA2, RNA3, and RNA4. P150 and RdRP, subunits of BNYVV RNA‐dependent RNA polymerase; RTD, read‐through protein. **(B)** Symptoms in systemically infected *B. macrocarpa* plants inoculated with BNYVV RNA1, 2, 3 co-infiltrated with RNA4^NM^, RNA4^XJ^, RNA4^O11^, or RNA4^OW1^ at 25 dpi. Scale bars, 2 cm. **(C)** Immunoblot analyzing accumulation of BNYVV coat protein (CP) and P31-FLAG in the samples of panel **(B)**. **(D)** RT–qPCR analyzing viral genomic RNA levels in the samples of panel **(B)**. The viral *CP* gene was used as an indicator of viral RNA levels. *Actin* was used as an internal control. Error bars indicate means ± SD of three biological repeats. Letters indicate significant differences (ANOVA, *P* < 0.05). **(E)** Above ground fresh weights of inoculated plants in panel **(B)**. **(F)** Symptoms of *N. benthamiana* leaves inoculated with BNYVV RNA1, 2 co-infiltrated with RNA4^NM^, RNA4^XJ^, RNA4^O11^, or RNA4^OW1^ at 10 dpi. Scale bars, 2 cm. **(G)** Accumulation of BNYVV CP and P31-FLAG in the samples of panel **(E)**, analyzed by immunoblotting. **(H)** Representative images of *N. benthamiana* inoculated by BNYVV RNA1, 2 co-infiltrated with RNA4^NM^, RNA4^XJ^, RNA4^O11^, or RNA4^OW1^ at 50 dpi. Scale bars, 5 cm. **(I)** Plant height in panel (G) was measured using ImageJ. **(J)** Phylogenetic analyses of P31 amino acids sequence in different RNA4 isolates. RNA4-encoded P31^NM^, P31^XJ^, P31^O11^, and P31^OW1^ were indicated by red. **(K)** Amino acid (aa) sequence alignment of aa 145-155 regions from P31^NM^, P31^XJ^, P31^O11^, and P31^OW1^. The non-conserved aa sequences were indicated by a red box. **(L)** Immunoblot analyzing P31 protein levels in *N. benthamiana* leaves expressing P31^NM^-FLAG, P31^O11^-FLAG, P31^XJ^-FLAG, and P31^OW1^-FLAG, respectively. In panels **(C)**, **(G)**, and **(L)**, RbcL served as loading control. Relative values (RV) of protein accumulation were analyzed according to band densities. In panels **(E)** and **(I)**, Error bar represents ± SD of 9 plants. Letters indicate significant differences (ANOVA, *P* < 0.05).

Genetic variation of BNYVV isolates in different geographical distributions is continuously enhancing viral virulence and leads to the emergence of resistance-breaking variants. BNYVV RNA4-encoded P31 is an important pathogenesis factor involved in virus transmission and RNA silencing suppression [24]. To explore the effect of genetic variation of BNYVV RNA4 on virus virulence, four RNA4 isolates, including two Chinese isolates from Inner Mongolia (RNA4^NM^) and Xin Jiang (RNA4^XJ^), as well as Japanese isolate O11 (RNA4^O11^) and Germany isolate OW1 (RNA4^OW1^) were cloned into the pBN4-FLAG plasmid, respectively (Figs 1A and S1A Fig).

Using the reverse genetics system of BNYVV [15], we co-infiltrated BNYVV RNA1, 2, 3 with RNA4^NM^, RNA4^XJ^, RNA4^O11^, or RNA4^OW1^ in *Beta macrocarpa* (*B. macrocarpa*) leaves (Figs 1A and S1A Fig). At 25 days post-infiltration (dpi), *B. macrocarpa* plants systemically infected with BNYVV^NM^ and BNYVV^XJ^ exhibited more severe symptoms, compared with those of BNYVV^O11^ and BNYVV^OW1^ (Fig 1B). Protein levels of BNYVV coat protein (CP) and P31-FLAG increased in *B. macrocarpa* plants infected BNYVV^NM^ and BNYVV^XJ^, compared to BNYVV^O11^ and BNYVV^OW1^ (Fig 1C). Moreover, reverse transcription quantitative polymerase chain reaction (RT–qPCR) showed that viral RNA levels were higher in plants infected with BNYVV^NM^ and BNYVV^XJ^, compared with BNYVV^O11^ and BNYVV^OW1^ (Fig 1D). The fresh weight of *B. macrocarpa* plants on above ground infected by BNYVV^NM^ and BNYVV^XJ^ was significantly lower than that of BNYVV^O11^ and BNYVV^OW1^ (Fig 1E).

To further confirm the pathogenicity of different RNA4 isolates, we further co-infiltrated BNYVV RNA1, 2 with RNA4^NM^, RNA4^XJ^, RNA4^O11^, or RNA4^OW1^ in *N*. *benthamiana* plants. At 10 dpi, BNYVV^NM^ and BNYVV^XJ^ causing severe crinkle leaf symptoms in systemically infected leaves compared with BNYVV^O11^ and BNYVV^OW1^ (Fig 1F). Immunoblotting and RT–qPCR showed that leaves systematically infected with BNYVV^NM^ and BNYVV^XJ^ contained higher accumulation of viral proteins and viral RNA, as compared to those of BNYVV^O11^ and BNYVV^OW1^ (Figs 1G and S1B). At 50 dpi, *N*. *benthamiana* plants systemically infected with BNYVV^NM^ and BNYVV^XJ^ was obviously shorter than those of BNYVV^O11^ and BNYVV^OW1^ (Fig 1H and Fig 1I). These results indicate that genetic variation of BNYVV RNA4 in different isolates affects virus virulence.

Phylogenetic analysis of P31 aa sequence indicated that P31^NM^ and P31^XJ^ belong to group I, while P31^O11^ and P31^OW1^ belong to group II (Fig 1J). Notably, the Arg-147 in P31^NM^ and P31^XJ^ was distinct from the Lys-147 in P31^O11^ and P31^OW1^ (Figs 1K and S2). Given the Lys residue is usually ubiquitinated by host E3 ligase for protein degradation, we speculate that the 147 residue regulates P31 stability. To this end, we infiltrated *N*. *benthamiana* leaves with RNA4^NM^, RNA4^XJ^, RNA4^O11^, or RNA4^OW1^. At 2 dpi, immunoblot results showed that protein levels of RNA4-encoded P31^NM^-FLAG and P31^XJ^-FLAG with Arg-147 were significantly higher than P31^O11^-FLAG and P31^OW1^-FLAG with Lys-147 (Fig 1L). These results suggest that the Lys-147 residue of P31^O11^ and P31^OW1^ cause protein instability and compromised virus virulence.

### Ubiquitination of the P31 Lys-147 residue negatively regulates protein stability

**Fig 2:**
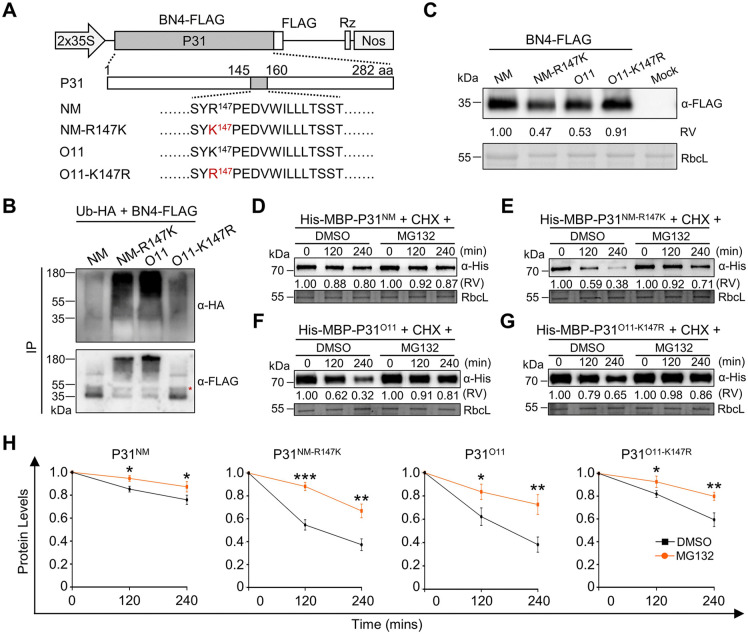
Ubiquitination of P31 Lys-147 negatively regulates protein stability. **(A)** Organization of the FLAG fused-P31^NM^, P31^O11^, and point mutants. **(B)** P31 ubiquitination levels in *N. benthamiana* leaves expressing Ub-HA with P31^NM^-FLAG, P31^NM-R147K^-FLAG, P31^O11^-FLAG, and P31^O11-K147R^-FLAG. Total proteins were immunoprecipitated with anti‐FLAG beads and blotted with anti-HA and anti-FLAG antibodies. The red asterisk represents weight chains. **(C)** Immunoblot analysis of P31-FLAG protein accumulation levels in *N. benthamiana* leaves inoculated with P31^NM^-FLAG, P31^NM-R147K^-FLAG, P31^O11^-FLAG, and P31^O11-K147R^-FLAG, respectively. **(D-G)** Cell-free degradation assays showed the degradation rate of P31^NM^ **(D)**, P31^NM-R147K^ **(E)**, P31^O11^ **(F)**, and P31^O11-K147R^ **(G)** in the presence or absence of MG132. Purified recombinant His-maltose binding protein (MBP)-P31, protein synthesis inhibitor cycloheximide (CHX, 0.5 mM), and ATP (20 mM) were incubated with total leaf protein extracts with DMSO or MG132 (50 μm) before sample collection at the indicated time points. **(H)** Normalized P31 degradation rates in panels **(D-G)**. Values are means ± SD of three independent repeats. *P < 0.05, **P < 0.01, and ***P < 0.001 (Student’s t test). In panels **(C–G)**, RbcL served as a loading control. Relative values (RV) of protein accumulation were analyzed according to band densities.

We next selected the P31^NM^ and P31^O11^ proteins to further explore the role of P31 Arg (R)/Lys (K)-147 in protein stability. To this end, the plasmids for expression of BN4^NM^, BN4^NM-R147K^, BN4^O11^, or BN4^O11-K147R^ (Fig 2A) were constructed and co-infiltrated with Ub-hemagglutinin (HA) into *N*. *benthamiana* leaves. At 2 dpi, infiltrated leaves were harvested and immunoblotted with anti-FLAG beads. Immunoblot analyses showed that ubiquitination levels of P31^O11^ and P31^NM-R147K^ were obviously higher than P31^O11-K147R^ and P31^NM^ (Fig 2B). Consistently, protein levels of P31^O11-K147R^ and P31^NM^ were higher than P31^O11^ and P31^NM-R147K^ in infiltrated *N*. *benthamiana* leaves (Fig 2C).

We then carried out cell-free protein degradation assays to monitor protein degradation rate of P31. Total protein extracts of *N*. *benthamiana* leaves were incubated with His-maltose binding protein (MBP)-P31, 26S-proteasome inhibitor MG132, protein synthesis inhibitor cycloheximide (CHX), and ATP. Compared with the DMSO treatment, MG132 treatment significantly inhibited degradation of P31^O11^ and P31^NM-R147K^ (Fig 2D–2H). These results indicate that the P31^O11^ Lys-147 residue is the main ubiquitinated site to induce protein instability, and that P31^NM^ at Arg-147 is resistant to protein degradation.

### Ubiquitination of P31^O11^ Lys-147 attenuates BNYVV infection

**Fig 3:**
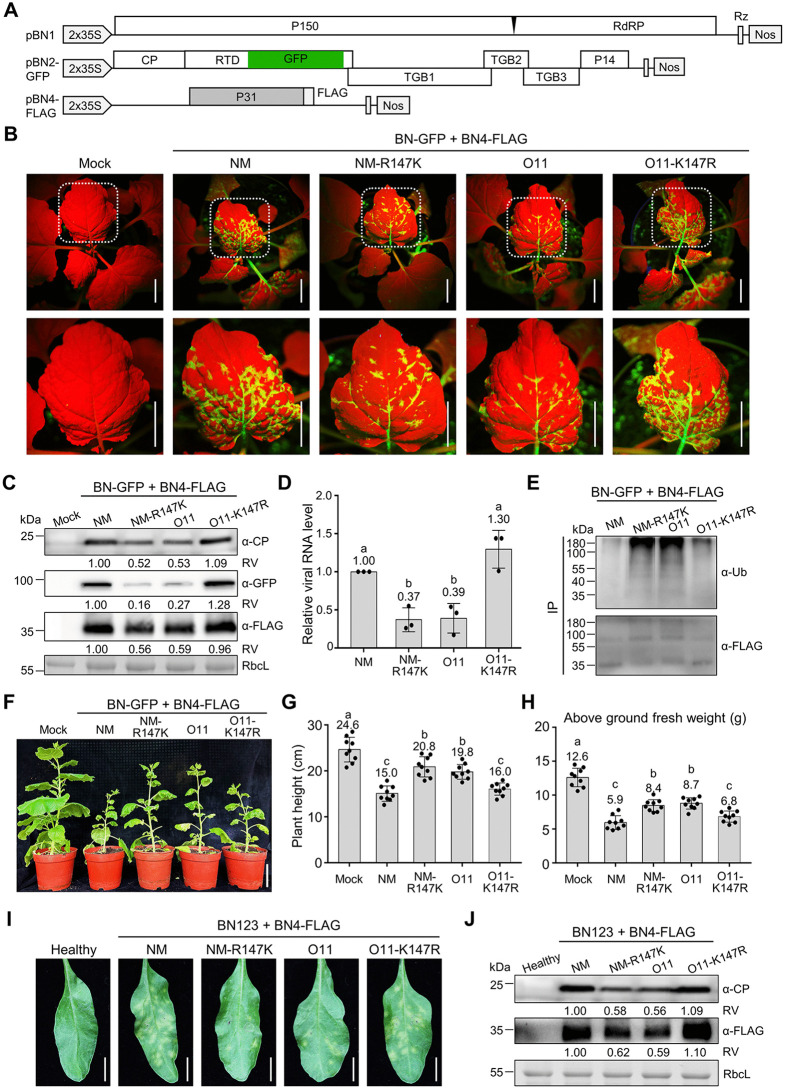
Ubiquitination of P31 at Lys-147 negatively regulates BNYVV infectivity. **(A) **Schematic representation of the pBN1, pBN2-GFP, and pBN4-FLAG constructs used to express BNYVV RNA1, RNA2-GFP, and RNA4.** (B)** GFP fluorescence in *N. benthamiana* leaves inoculated with BNYVV RNA1, 2-GFP (BN-GFP) co-expressed with RNA4^NM^, RNA4^NM-R147K^, RNA4^O11^, or RNA4^O11-K147R^ at 10 dpi. Scale bars, 2 cm. **(C)** Immunoblot analyzing accumulation of BNYVV CP, GFP, and P31-FLAG in the samples of panel (A) using the antibodies as indicated. **(D)** RT–qPCR analyzing viral RNA accumulation in the samples of panel **(B)** using the *CP* gene to represent viral RNA levels. *EF1α* was used as an internal control. Error bars indicate means ± SD of three biological repeats. Letters indicate significant differences (ANOVA, *P* < 0.05). **(E)** P31 ubiquitination levels during BNYVV infection in the samples of panel **(B)**. Total protein extracts were immunoprecipitated with anti-FLAG beads at 10 dpi, followed by immunoblot analysis with the indicated antibodies. **(F)** Representative images of *N. benthamiana* plants inoculated with BN-GFP co-expressed with RNA4^NM^, RNA4^NM-R147K^, RNA4^O11^, or RNA4^O11-K147R^ at 35 dpi. Scale bars, 5 cm. **(G)** Plant height were measured using ImageJ for panel **(F)**. **(H)** Above ground fresh weights of inoculated plants in panel **(F)**. **(I)** Symptoms in infected *B. vulgaris* leaves inoculated with BNYVV RNA1, 2, 3 co-expressed with RNA4^NM^, RNA4^NM-R147K^, RNA4^O11^, or RNA4^O11-K147R^ at 15 dpi. Scale bars, 1 cm. **(J)** Immunoblot analyzing protein levels of BNYVV CP and P31-FLAG in the samples of panel **(I)**. In panels **(C)** and **(J)**, RbcL served as a loading control. Relative values (RV) of protein accumulation were analyzed according to band densities. In panels **(G)** and **(H)**, Error bar represents ± SD of 9 plants. In panels **(D)**, **(G)**, and **(H)**, letters indicate significant differences (ANOVA, *P* < 0.05).

We previously obtained the recombinant BNYVV RNA1, RNA2-GFP (hereafter called BN-GFP) infectious clone carrying a *green fluorescent protein* (*GFP*) gene to monitor virus infection [15]. To investigate the biological function of P31 Lys/Arg-147, BN-GFP was co-expressed with RNA4^NM^, RNA4^NM-R147K^, RNA4^O11^, or RNA4^O11-K147R^ in *N*. *benthamiana* plants (S1A Fig). At 10 dpi, leaves systemically infected with BNYVV^O11^ exhibited decreasing GFP fluorescence intensity, accompanied with reduced virus protein and virus RNA levels, as compared to those of the point mutation BNYVV^O11-K147R^ (Fig 3A–Fig 3D). Correspondingly, ubiquitination levels of P31^O11^ were higher than P31^O11-K147R^ (Fig 3E). At 35 dpi, the plant height and above ground fresh weight of plants systemically infected with BNYVV^O11-K147R^ were significantly lower than that of BNYVV^O11^ (Fig 3F–Fig 3H). These results indicate that the BNYVV^O11^ has less virulence than the point mutation BNYVV^O11-K147R^. Conversely, the BNYVV^NM^ exhibited higher virulence than the point mutation BNYVV^NM-R147K^ (Fig 3B–Fig 3H).

In addition, *B. vulgaris* leaves were co-infiltrated BNYVV RNA1, 2, 3 with RNA4^NM^, RNA4^NM-R147K^, RNA4^O11^, or RNA4^O11-K147R^. At 15 dpi, more yellow lesions and viral protein accumulation were present in *B. vulgaris* leaves infected with BNYVV^NM^ and BNYVV^O11-K147R^ compared with those of BNYVV^O11^ and BNYVV^NM-R147K^ (Fig 3I and Fig 3J). Collectively, these results suggest that ubiquitination of the P31 Lys-147 residue on P31^O11^ and P31^NM-R147K^ negatively regulates BNYVV infection.

### P31 is a substrate of the E3 ligase HRD1a

**Fig 4:**
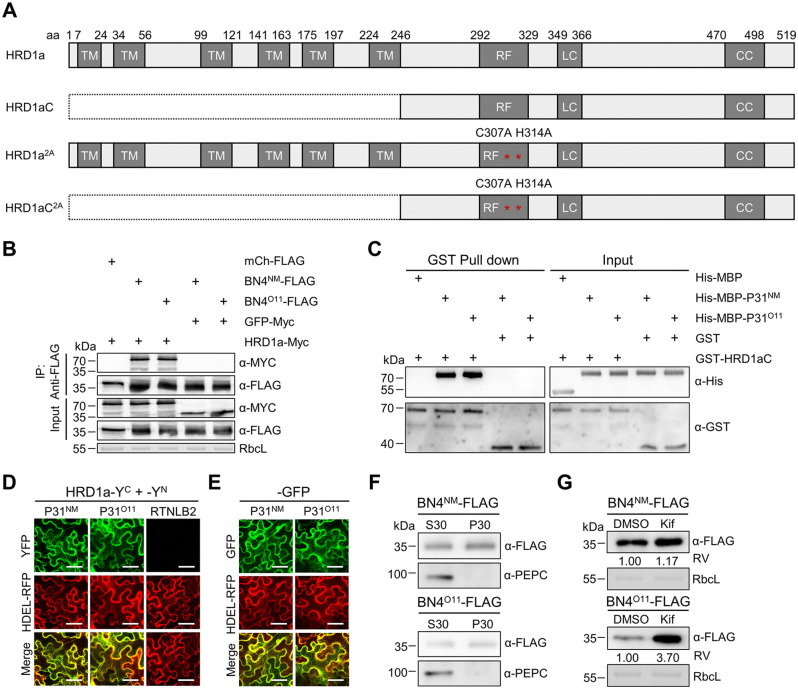
The BNYVV P31 interacts with E3 ligase NbHRD1a. **(A)** Schematic representation of HRD1a and its deletion mutant. CC, coiled-coil domain; LC, low-complexity domain; RF, RING finger domain; TM, trans-membrane domain. Red asterisks represent E3 ligase activity mutation sites. **(B)** Co-immunoprecipitation (Co-IP) analysis of interactions between HRD1a and P31 in *N. benthamiana* leaves. Leaves were agroinfiltrated with various combinations of constructs and then harvested at 2 dpi. Total proteins were immunoprecipitated with anti‐FLAG beads. mCherry (mCh)-FLAG and GFP-Myc served as negatively controls. **(C)** Glutathione S-transferase (GST) pull‐down assay examining interactions between HRD1a and P31 interaction *in vitro*. Pull-down and input products were analyzed by immunoblot with anti-His or anti-GST antibodies. His-MBP protein and GST protein served as negative controls. **(D)** Bimolecular fluorescence complementation (BiFC) assay analyzing the HRD1a and P31 interaction *in vivo*. The yellow fluorescent protein (YFP) signal was visualized by confocal microscopy at 2 dpi. RTNLB2 served as a negative control. **(E)** Confocal microscopy imaging showing co-localization of P31^NM^-GFP and P31^O11^-GFP with RFP-HDEL. **(F)** Cell fractionation assays showed the P31 protein in the membrane-enriched fraction (P30) and the soluble fraction (S30). PEPC was used as cytosolic marker. **(G)** Immunoblot analyzing P31-FLAG accumulation in leaves of *N. benthamiana* treated with DMSO or kifunensine (Kif). P31-FLAG transiently expressed in *N. benthamiana* leaves in the presence of Kif (50 μM) or DMSO. At 2 api, Total protein extracts were subjected to SDS-PAGE and immunoblotting using anti-FLAG antibody. Relative values (RV) of protein accumulation were analyzed according to band densities. In panels **(B)** and **(G)**, RbcL served as loading controls. In panels **(D)** and **(E)**, HDEL-RFP served as a cortical ER marker. Scale bars, 50 μm.

To explore the potential E3 ligases responsible for P31 ubiquitination, we performed split-TurboID-based proximity labeling assays to screen for the interacting candidates of P31 (S3 Fig). The E3 ligase HRD1a was identified as a putative P31 interacting candidate in *N. benthamiana* plants. *HRD1a* (*Nbe06g19750.1*, see http://lifenglab.hzau.edu.cn/Nicomics/) encodes a protein consisting of 530 aa, which contains four domains, including six transmembrane domains, a RING finger domain, a low-complexity domain, and a coiled-coil domain (Fig 4A). This gene shares more than 95% nucleotide sequence identity with *Nbe05g21720.1* (see http://lifenglab.hzau.edu.cn/Nicomics/), which was temporarily named as *HRD1b*.

To validate the P31-HRD1a interaction *in vivo*, we carried out co‐immunoprecipitation (Co‐IP) assays through co-infiltration of HRD1a-Myc with P31^NM^-FLAG or P31^O11^-FLAG. At 2 dpi, total plant proteins were extracted for immunoprecipitation with anti-FLAG affinity beads. Immunoblot analyses showed that both P31^NM^-FLAG and P31^O11^-FLAG were co-immunoprecipitated with HRD1a (Fig 4B). The interactions of the controls including GFP-Myc or mCh-FLAG were negative (Fig 4B).

We further performed the glutathione S-transferase (GST) pull-down assay to test the interaction of P31 and HRD1a *in vitro*. Since the full-length HRD1a with N-terminal transmembrane domains (1–246 aa) was hardly purified, the C-terminal fragment of HRD1a (247–530 aa) was fused with a GST tag (GST-HRD1aC) and purified form *Escherichia coli* cells (Fig 4A). Then, GST-HRD1aC was incubated with His-MBP-P31^NM^ or His-MBP-P31^O11^ for pull-down with anti-GST beads. Immunoblot results showed that GST-HRD1aC interacted with His-MBP-P31^NM^ or His-MBP-P31^O11^, while the GST control did not (Fig 4C). Additionally, GST-HRD1aC failed to be pulled down with the His-MBP protein (Fig 4C).

Then we carried out the bimolecular fluorescence complementation (BiFC) assay to confirm the interaction of P31-HRD1a *in vivo*. P31^NM^-Y^N^ or P31^O11^-Y^N^ was co-expressed with HRD1a-Y^C^ in *N. benthamiana* leaves by agroinfiltration, respectively. At 2 dpi, strong yellow fluorescent protein (YFP) signal was observed in *N. benthamiana* leaves expressing HRD1a-Y^C^ with P31^NM^-Y^N^ or P31^O11^-Y^N^ (Fig 4D). Besides, the BiFC signal was co-localized with the ER marker, HDEL-RFP, indicating that the interaction occurred in the ER. The negative control RETICULON-LIKE PROTEIN B2-Y^N^ [39] did not interact with HRD1a-Y^C^ (Fig 4D), despite all proteins were successfully expressed in leaves (S4 Fig). Collectively, these results indicate that BNYVV P31 interacts with HRD1a in *vivo* and in *vitro*.

Given that the P31–HRD1a interaction *in vivo* and *in vitro*, we next tested whether P31 is a HRD1a substrate and degraded by the related ERAD pathway. Confocal microscopy and cell fractionation assays showed that both P31^NM^ and P31^O11^ were localized in the ER (Fig 4E–4F). We next co-infiltrated 50 μM ERAD inhibitor kifunensin (Kif) with P31^NM^-FLAG or P31^O11^-FLAG. Immunoblot showed that the Kif treatment significantly increased the protein levels of the P31^O11^-FLAG compared to DMSO (Fig 4G). In addition, the Kif treatment only slightly increased P31^NM^-FALG (Fig 4G). These results indicate that P31 interacts with HRD1a as an ERAD substrate.

### HRD1a mainly ubiquitinates P31 Lys-147 for protein degradation

**Fig 5:**
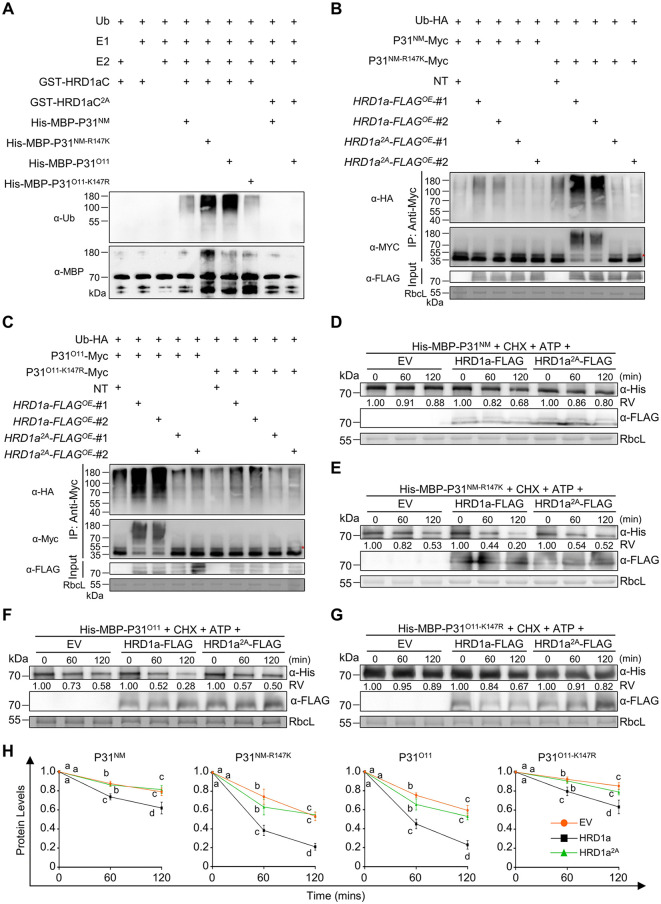
NbHRD1a ubiquitinates P31^O11^ Lys-147 and reduces its protein levels. **(A)** Immunoblot analysis of the effect of HRD1a on P31 ubiquitination *in vitro*. The reaction system comprised Triticum aestivum E1 (GenBank: GI: 136632), Homo sapiens E2 (GenBank: UBCh5b), GST-HRD1aC, and His-MBP-P31 at 37°C for 1 h**.** GST-HRD1aC^Cys307Ala/His312Ala^ (HRD1aC^2A^) was served as a negative control. Ubiquitination of P31 was detected with anti-MBP antibody. Ubiquitinated proteins were immunoblotted with anti-ubiquitin antibodies. **(B)** Immunoblot analysis of the effect of HRD1a on P31^NM^ and P31^NM-R147K^ ubiquitination in transgenic *N. benthamiana* plants. **(C)** Immunblot analysis of the effect of HRD1a on P31^O11^ and P31^O11-K147R^ ubiquitination in transgenic *N. benthamiana* plants. **(D-G)** Cell-free degradation assays showed the degradation rate of P31^NM^ **(D)**, P31^NM-R147K^ **(E)**, P31^O11^ **(F)**, and P31^O11-K147R^ **(G)** in the presence of HRD1a or HRD1a^Cys307Ala/His312Ala^ (HRD1a^2A^). Recombinant purified MBP-P31 protein incubated with total leaf protein extracts was infiltrated with HRD1a, HRD1a^2A^, or EV. Samples incubated for the indicated time points with CHX and ATP, immunoblotting analysis with anti-His and anti-FLAG antibody. **(H)** Normalized P31 degradation rate experiments in panels **(D-G)**. Values are means ± SD of three independent repeats. Different letters indicate significant differences (analysis of variance, *P* < 0.05). In panels **(B)** and **(C)**, Non-transgenic (NT), overexpressing *HRD1a*-*FLAG* or overexpressing *HRD1a^2A^*-*FLAG* transgenic *N. benthamiana* plants co-expressed P31-Myc and Ub-HA. Total protein extracts were immunoprecipitated with anti-MYC beads, followed by immunoblot analysis with the indicated antibodies. Red asterisks represent weight chains. In panels **(B-G)**, RbcL served as loading control. In panels **(D-G)**, relative values (RV) of protein accumulation were analyzed according to band densities.

We then performed *in vitro* assays to verify whether HRD1a directly ubiquitinates P31. The E1 (GenBank: GI: 136632), E2 (GenBank: UBCh5b), Ub, GST-HRD1aC, and His-MBP-P31 proteins were incubated at 37°C for 60 mins. The point mutation GST-HRD1aC^Cys307Ala/His312Ala^ (HRD1aC^2A^) without the E3 ligase activity [36] served as a negative control. Immunoblot showed that HRD1aC induced high ubiquitination signal on P31^O11^ and P31^NM-R147K^ *in vitro*, but very faint signals on P31^NM^ and P31^O11-K147R^. Additionally, HRD1aC^2A^ could not ubiquitinate P31^NM^ and P31^O11^ *in vitro* (Fig 5A).

We next explored the role of HRD1a in P31 ubiquitination *in vivo*. Firstly, we proved that P31^O11^-FLAG is ubiquitinated in *N. benthamiana* leaves, rather than mCh-FLAG (S5A Fig). Then, *N. benthamiana* leaves were co-infiltrated Ub-HA, P31-Myc with HRD1a-FLAG or HRD1a^2A^-FLAG. At 2 dpi, immunoblot with anti-MYC beads showed that leaves expressing HRD1a-FLAG improved ubiquitination levels of the P31^O11^ and P31^NM-R147K^, but not on those of P31^NM^ and P31^O11-K147R^ (S5B Fig). Moreover, *N. benthamiana* leaves expressing HRD1a^2A^-FLAG did not change ubiquitination levels of P31^NM^ and P31^O11^ (S5B Fig). These results indicate that HRD1a mainly mediates ubiquitination of the P31 Lys-147 *in vitro* and *in vivo*.

We have generated overexpression lines of *HRD1a*-*FLAG* (*HRD1a*-*FLAG*^OE^ #1 and #2) and *HRD1a*^*2A*^-*FLAG* (*HRD1a*^*2A*^-*FLAG*^OE^ #1, #2, #3, and #4) in our previous study [36]. To test the influence of HRD1a on P31 ubiquitination in transgenic *N. benthamiana* plants, non-transgenic (NT), two *HRD1a*-*FLAG*^OE^ lines and two *HRD1a*^*2A*^-*FLAG*^OE^ lines were infiltrated to co-express Ub-HA and P31-Myc. Immunoblotting results showed that P31^NM-R147K^ ubiquitination levels were significantly enhanced in two *HRD1a*-*FLAG*^OE^ lines compared with those of the P31^NM^ (Fig 5B). In contrast, the P31^O11-K147R^ ubiquitination levels were significantly lower in two *HRD1a*-*FLAG*^OE^ lines, compared with the P31^O11^ (Fig 5C). Furthermore, ubiquitination levels of the P31^NM^ and P31^O11^ were lower in two *HRD1a*^*2A*^-*FLAG*^OE^ lines than in two *HRD1a*-*FLAG*^OE^ lines (Fig 5B and 5C). These results suggest that HRD1a mediates ubiquitination of the P31 Lys-147 in transgenic *N. benthamiana* plants.

We next carried out cell free assays to validate whether HRD1a negatively regulated the P31 levels. *N. benthamiana* leaves were infiltrated with HRD1a-FLAG or HRD1a^2A^-FLAG. At 2dpi, the His-MBP-P31 protein was incubated with ATP, CHX, and total leaf extracts of HRD1a-FLAG or HRD1a^2A^-FLAG. Immunoblot showed that degradation rates of the P31^O11^ and P31^NM-R147K^, rather than the P31^NM^ and P31^O11-K147R^, were significantly reduced in the presence of HRD1a-FLAG (Fig 5D–5H). Besides, HRD1a^2A^-FLAG did not affect the degradation rate of P31 (Fig 5D–5H). Collectively, HRD1a recognizes P31 Lys-147 and ubiquitinates it to trigger protein degradation.

### P31^NM^ with Arg-147 evades from HRD1a-mediated antiviral defense

**Fig 6:**
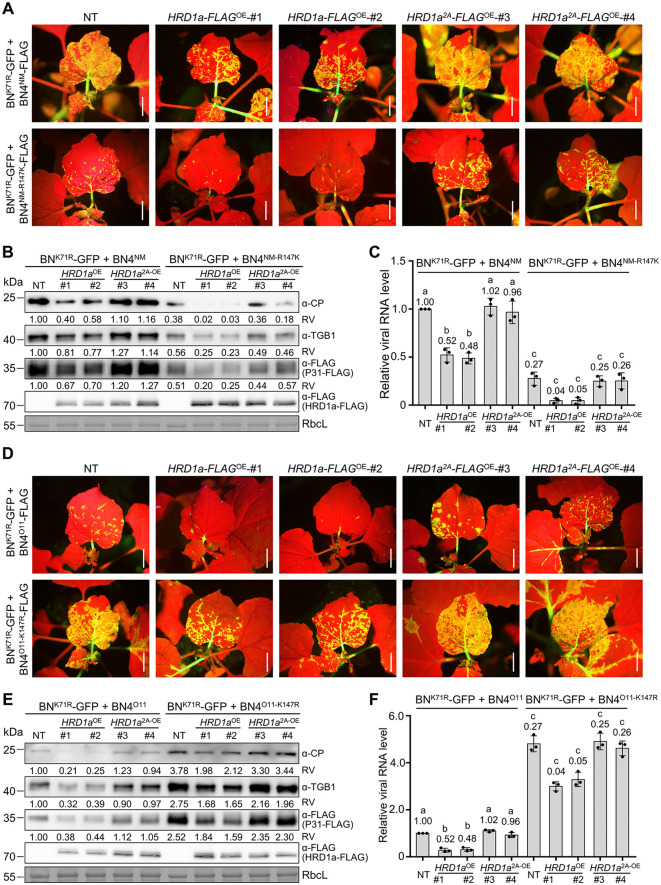
NbHRD1a inhibits BNYVV infection in transgenic *N. benthamiana* plants. **(A)** GFP fluorescence in NT, *HRD1a-FLAG*^OE^, and *HRD1a*^*2A*^*-FLAG*^OE^ plants infected by BN^K71R^-GFP with RNA4^NM^ or RNA4^NM-R147K^ at 11 dpi. Scale bars, 2 cm. **(B)** Immunoblot analyzing protein levels of BNYVV CP, TGB1, and FLAG-tagged proteins in leaf samples of **(A)**. **(C)** RT–qPCR analyzing viral genomic RNA levels in the samples of **(A)**. **(D)** GFP fluorescence in NT, *HRD1a-FLAG*^OE^, and *HRD1a*^*2A*^*-FLAG*^OE^ plants infected by BN^K71R^-GFP with RNA4^O11^ or RNA4^O11-K147R^ at 11 dpi. Scale bars, 2 cm. **(E)** Immunoblot analyzing protein levels of BNYVV CP, TGB1, and FLAG-tagged proteins in the leaf samples of **(D)**. **(F)** RT–qPCR analyzing viral genomic RNA levels in the samples of **(D)**. In panels **(B)** and **(E)**, RbcL served as loading control. Relative values (RV) of protein accumulation were analyzed according to band densities. In panels **(C)** and **(F)**, *EF1α* served as an internal reference. The *CP* gene was used as the indicator of viral RNA levels. Values are means ± SD of three independent repeats. Different letters indicate significant differences (analysis of variance, *P* < 0.05).

Previously, we have proven that HRD1a ubiquitinates and reduces protein level of BNYVV triple gene block1 (TGB1) movement protein, while does not ubiquitinate and decrease protein levels of TGB1^K71R^. Moreover, the BN-GFP mutant containing TGB1^K71R^ (BN^K71R^-GFP) exhibits higher infectivity than BN-GFP [36]. To reduce the influence of HRD1a ubiquitinates TGB1, we infiltrated NT, two *HRD1a*-*FLAG*^OE^ lines and two *HRD1a*^*2A*^-*FLAG*^OE^ lines with BN^K71R^-GFP and RNA4. At 11 dpi, systemically infected leaves of two *HRD1a*-*FLAG*^OE^ lines infected with BNYVV^NM-R147K^ exhibited decreased GFP fluorescence intensity, virus protein levels, and virus RNA levels compared to BNYVV^NM^ (Fig 6A–6C). Furthermore, BNYVV^O11-K147R^ exhibited increased infectivity on *HRD1a*-*FLAG*^OE^ lines compared with BNYVV^O11^ (Fig 6D–6F). Additionally, *HRD1a*^*2A*^-*FLAG*^OE^ lines did not show obvious differences compared to NT plants after being challenged with BNYVV^NM^, BNYVV^O11^, BNYVV^NM-R147K^, and BNYVV^O11-K147R^ (Fig 6). Collectively, these results indicate that infection of BNYVV^O11^ isolate with P31 Lys-147 is suppressed by HRD1a, whereas the BNYVV^NM^ isolate with P31 Arg-147 escapes from HRD1a inhibition.

### Knockout of *HRD1* reduces P31 ubiquitination and promotes BNYVV infection in *N. benthamiana* plants

**Fig 7:**
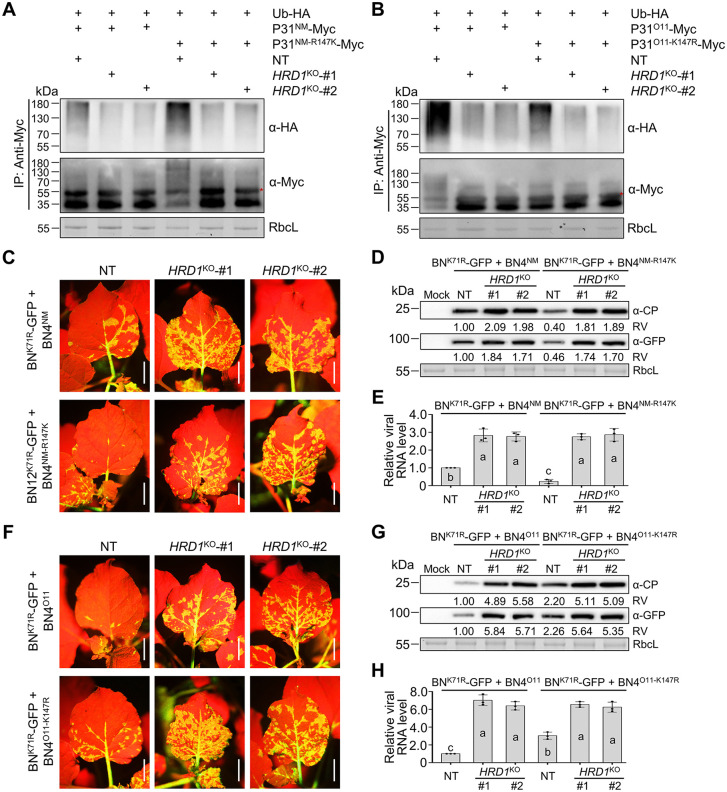
Knockout of *NbHRD1* decreases P31 ubiquitination and promotes BNYVV infection. **(A–B)** Immunoblot analyzing the effect of HRD1 on P31^NM^, P31^NM-R147K^, P31^O11^, and P31^O11-K147R^ ubiquitination in *NbHRD1*^KO^ transgenic plants. NT or *NbHRD1*^KO^ transgenic plants co-expressed P31-Myc and Ub-HA. Total protein extracts were immunoprecipitated with anti-MYC beads, followed by immunoblot analyses with indicated antibodies. Red asterisks represent weight chains. **(C)** GFP fluorescence in NT and *NbHRD1*^KO^ plants infected by BN^K71R^-GFP with RNA4^NM^ or RNA4^NM-R147K^ at 9 dpi. Scale bars, 2 cm. **(D)** Immunoblot analyzing protein levels of BNYVV TGB1 and FLAG-tagged proteins in the leaf samples of **(B)**. Relative values (RV) of protein accumulation were analyzed according to band densities. **(E)** RT–qPCR analyzing viral genomic RNA levels in the samples of **(B)**. The *CP* gene was used as the indicator of viral RNA levels. **(F)** GFP fluorescence in NT and *NbHRD1*^KO^ plants infected by BN^K71R^-GFP with RNA4^O11^ or RNA4^O11-K147R^ at 9 dpi. Scale bars, 2 cm. **(G)** Immunoblot analyzing protein levels of BNYVV TGB1 and FLAG-tagged proteins in the leaf samples of **(F)**. Relative values (RV) of protein accumulation were analyzed according to band densities. **(H)** RT–qPCR analyzing viral genomic RNA levels in the samples of **(F)**. In panels **(E)** and **(H)**. The *CP* gene was used as the indicator of viral RNA levels. Values are means ± SD of three independent repeats. Different letters indicate significant differences (analysis of variance, *P* < 0.05).

We further used the clustered regularly interspaced short palindromic repeats (CRISPR)-associated nuclease 9 (Cas9)-mediated genome editing to generate two knockout lines of *HRD1* (*HRD1*^KO^ #1 and #2). Notably, the guide RNA was chosen from nt 108–127 region downstream of the HRD1a start codon, which is a conserved region of *HRD1a* and *HRD1b* (S6 Fig). In the *HRD1*^KO^ lines, insertion or deletion mutations occurred in the CRISPR/Cas9‐targeted regions of *HRD1a* and *HRD1b* (S7A and S7B Fig). Two *HRD1*^KO^ lines exhibited developmental phenotypes similar to those of NT plants (S7C–S7E Fig). Then, Ub-HA and P31-Myc were co-expressed in two *HRD1*^KO^ lines and NT plants. Immunoblotting results showed decreased ubiquitination levels of P31^NM^-Myc, P31^NM-R147K^-Myc, P31^O11^-Myc, and P31^O11-K147R^-Myc in two *HRD1*^KO^ lines, compared to that of NT plants (Fig 7A and 7B). The P31^NM^ and P31^O11-K147R^ still exhibited minor ubiquitination (Fig 7A and 7B), indicating that other sites of P31^NM^ and P31^O11-K147R^ may be ubiquitinated by other host E3 ligases.

We then challenged NT or two *HRD1*^KO^ lines with BN^K71R^-GFP and RNA4. At 9 dpi, when compared to NT plants, systemically infected leaves in two *HRD1*^KO^ lines infected with BNYVV^NM^, BNYVV^O11^, or virus mutants showed significantly enhanced GFP fluorescence intensity, viral protein, and viral RNA levels (Fig 7C–7H). Collectively, these results indicate that decreased P31 ubiquitination levels promote BNYVV infection in transgenic *HRD1*^KO^ plants.

**Fig 8:**
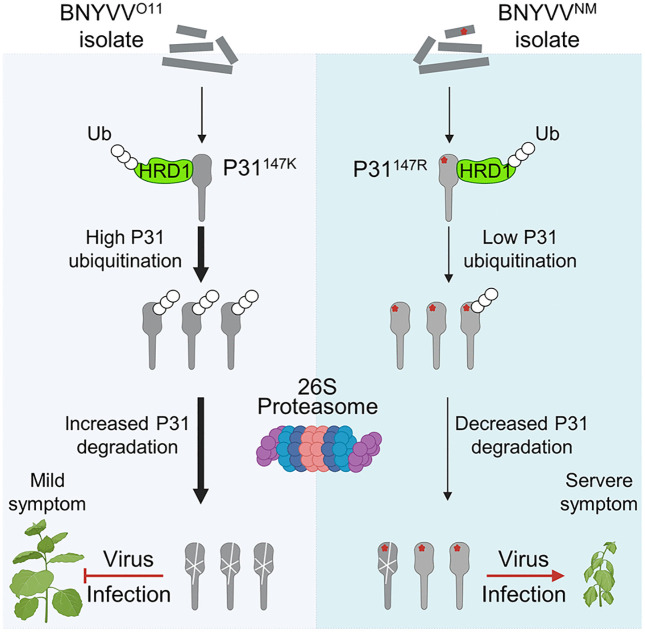
A proposed model illustrating how BNYVV evades HRD1-mediated antiviral defense. BNYVV P31 is translated from the viral genome and interacts with the E3 ligase HRD1. HRD1 increases BNYVV RNA4^O11^-encoded P31^K147^ ubiquitination levels to trigger 26S proteasome-mediated degradation for inhibiting virus infection in plants. However, BNYVV RNA4^NM^-encoded P31^R147^ was insensitive for HRD1 triggering the ubiquitination and 26S proteasome-mediated degradation, thus promoting viral infectivity in plants. Red asterisks represent the P31 Arg-147 residue of RNA4^NM^. This Figure was created with BioRender.com (https://www.biorender.com/). Created in BioRender. Gao, Q. (2026) https://BioRender.com/su3kcv3ssss.

In summary, our results reveal that the E3 ligase HRD1 inhibits BNYVV infection by triggering ubiquitination and degradation of the BNYVV^O11^-encoded P31^147K^. However, the natural variant BNYVV^NM^-encoded P31^147R^ evades HRD1-mediated ubiquitination and degradation through the 26S proteasome, resulting in higher infectivity in plants (Fig 8). These results provide new evidence showing the co-evolutional arms race between viruses and host plants.

## Discussion

Virus diseases cause huge economic losses to global agriculture annually [40]. BNYVV is a significant pathogen in *Beta* genus crops and possesses notable genetic and phenotypic diversity [18,24,25]. The BNYVV isolates harboring different combinations of genomic RNAs significantly affect symptom development and disease severity [27,41]. BNYVV RNA3-encoded P25 is a well-characterized pathogenicity determinant, and its sequence variations influence symptoms expression on *Chenopodiace quinoa*, *Tetragonia expansa* and *B. vulgaris* species [20,42]. Comprehensive sequence analysis reveals that the 67^th^-70^th^ residues of P25 are highly variable and subjected to strong positive selection [17,18]. Furthermore, natural mutations of P25 at the amino acids of 67 and 68 generate resistance-breaking isolates in Europe, the United States, and China [18,42,43].

BNYVV RNA4-encoded P31 is responsible for *P. betae* transmission, silencing suppression in root and symptom severity on *Tetragonia expansa* and *N. benthamiana* plants [26,27]. Although 264 BNYVV RNA 4 sequences from different countries have been deposited in NCBI database (S1 Table), how natural variation of P31 affects viral pathogenesis on *Beta* genus crops and host defense still remains largely unknown. Here, we found that BNYVV isolates show different virulence attributed to different groups of *P31* gene from four geographical locations (Fig 1). BNYVV isolates NM and XJ induce more severe dwarf symptoms than isolates O11 and OW1 in *B. macrocarpa* and *N. benthamiana* plants (Fig 1). Phylogenetic analysis of P31 aa sequences showed P31^NM^ and P31^XJ^ belong to group I, while P31^O11^ and P31^OW1^ fall into group II (Fig 1). Previous studies have indicated that RNA3-encoded P25, rather than RNA4-encoded P31, is the major virulence factor affecting foliar and root symptoms of sugar beet plants [25,44–46]. The conclusion is based on group II isolates of BNYVV RNA4 like P31^O11^ that is easily targeted for degradation [25,41]. However, the increased stability of P31^NM^ and P31^XJ^ in group I causes severe symptoms in *B. macrocarpa* plants (Fig 1), highlighting that P31 is also an important virulence factor during BNYVV infection. Thus, the significant effect of BNYVV RNA4 in disease development of sugar beet plants might be underestimated. Investigation of P31 functions will enhance our understanding of BNYVV pathogenesis in future studies.

Plants exploit various mechanisms to defend themselves against virus infections [40]. The UPS system is one of the most efficient antiviral defense strategies [47]. Recent studies show that the UPS components or regulators directly target and ubiquitinate viral proteins for degradation or recognize viral protein to trigger downstream antiviral responses [32,36,48]. Our previous studies show that the UPS system is induced by BNYVV infection [27] and further demonstrate that the E3 ligase HRD1a of ERAD enhances plant antiviral immunity by ubiquitinating viral movement proteins [36]. Here, we discovered that HRD1a also interacted with viral virulence factor P31 in the ER (Fig 4). Furthermore, HRD1 inhibits BNYVV infection and alleviates symptoms by triggering ubiquitination and degradation of the P31^O11^ through the ERAD pathway (Figs 5 and 6). These results emphasize that the ubiquitin proteasome-dependent ERAD pathway targets various viral proteins and plays a critical role in plant antiviral immunity.

To counter defense against host UPS-mediated antiviral pathway, some viral proteins can suppress enzyme activities of host E3 ligases or utilize host UPS to degrade resistance proteins [3,37,49]. For instance, the P5-1 protein of *Spinareoviridae alporyzae* (rice black-streaked dwarf virus) can inhibit enzyme activity of the E3 ligase S-phase kinase-associated protein 1 (SKP1)-cullin 1 (CUL1)-F-box protein [37]. Moreover, *Tenuivirus oryzaclavatae* (rice stripe virus)-encoded P2 utilizes host UPS for non-expressor of pathogenesis-related genes 1 degradation to enhance virus infection [49]. However, little is known about how viruses-encoded proteins escape the UPS-mediated antiviral defense. In this study, the P31^O11-K147^ was certainly ubiquitinated by HRD1a, whereas P31^NM-R147^ was less insensitive to HRD1a-mediated ubiquitination (Figs 5 and 6). In addition, the K147R point mutation of P31^O11^ was rescued to the insensitivity to HRD1a-mediated ubiquitination, leading to severe symptoms (Fig 6). Moreover, the site-specific selection pressure analysis indicated that the amino acid position at 147 residue of 264 isolates (S1 Table) was rated as under negative purifying selection (dN/dS = 0.10) by the SLAC (Single-likelihood Ancestor Counting) method (see http://www.datamonkey.org). These results suggest that BNYVV genomic mutations result in the emergence of new natural variants to evade the UPS-mediated host antiviral immunity. Notably, although P31 has evaded HRD1-mediated ubiquitination through K147R mutation, P31^NM^ and P31^O11-K147R^ still exhibits little ubiquitination (Fig 7), indicates that other sites of P31^NM^ and P31^O11-K147R^ may be ubiquitinated by other host E3 ligases, an interesting question to be investigated in future studies.

Co-evolutionary events between viruses and host plants occur continuously [50]. During the ongoing arm races, host plants employ multiple layers of defense mechanisms to fight against viral infections, including innate immunity, RNA silencing, *R*-gene-mediated resistance, UPS and autophagy-mediated degradation systems [51–54]. In turn, plant viruses encode various pathogenic factors to counter defense [55–57]. For instance, the non-structural protein (NSs) of *Orthotospovirus tomatomaculae* (tomato spotted wilt virus, TSWV) facilitates virus infection through suppressing host antiviral RNA silencing [58]. Host plants evolve the *tomato spotted wilt* (*Tsw*) resistant gene to recognize NSs and trigger hypersensitive response for plant immunity [59]. To counter defense, naturally resistance-breaking (RB) TSWV strains produce a single point mutation in the NSs to evade *Tsw-*mediated resistance [60]. The *Tobacco mosaic virus resistance-2*^*2*^ (*Tm-2*^*2*^) gene confers tomato plant extreme resistance against tobamoviruses including *Tobamovirus tabaci* (tobacco mosaic virus, TMV) and *T. tomatotessellati* (tomato mosaic virus, ToMV) by recognizing the avirulence (Avr) viral MP for over sixty years. However, *T. fructirugosum* (tomato brown rugose fruit virus, ToBRFV) has broken resistance in tomato varieties due to MP variations [61,62]. Similarly, BNYVV P31 is also an important key virulence factor and silencing suppressor, but P31 is targeted for degradation by the host UPS pathway. Meanwhile, natural genetic variation of a single amino acid of P31 protein confers it ability to evade host antiviral ubiquitin/proteasome defense mechanism (Figs 5 and 6). Interestingly, among the four groups of BNYVV P31 isolates, all members of group I encode the highly virulent P31^R147^, while the remaining isolates of groups II, III, and IV encode P31^K147^ targeted by HRD1 [18]. BNYVV P31 isolates of group II and III distribute worldwide, while members of group IV occur in German, France and China. Recent studies (2014–current) have shown that all BNYVV RNA4 isolates in China belong to group I with the highly virulent P31^R147^. However, earlier studies (1992–2007) detect majority of Chinese RNA4 isolates encode P31^R147^, and only a few isolates encode P31^K147^, including the CY3 isolate (1992), CX2 isolate (2000) and two isolates Har4 and Bao (2007) [17,18,21]. These results suggest that BNYVV highly virulent strains with P31^R147^ has become dominant strains in China. Notably, one isolate UK-FF from United Kingdom in 2007 and seven Japanese isolates identified during 1991–2010 encode highly virulent P31^R147^ [17,20], indicating the possible appearance of some highly pathogenic BNYVV strains in these regions. Thus, continuous monitoring of genetic diversity of BNYVV P31 population is urgently needed.

In conclusion, we reveal the mechanism of BNYVV P31 natural variation evading HRD1-triggered protein degradation (Fig 8). In the proposed model, the E3 ligase *HRD1* targets BNYVV P31 in the ER membrane. Moreover, the Lys-147 of P31^O11^ is mainly targeted by HRD1 for ubiquitination and protein degradation. In contrast, the P31^NM^ Arg-147 is resistant to HRD1-triggered degradation, thereby evading HRD1-mediated protein degradation and promoting viral infectivity. This study provides evidence for continuous natural virus evolution and disease induction. Attention is needed to monitor genetic diversity of BNYVV P31 in future studies to forecast disease severity.

## Materials and methods

### Plasmid constructions

The cDNA of *P31*^NM^ or *P31*^O11^ was amplified from plasmids of pBN4^NM^ or pBN4^O11^, and recombined into the pGD-6xMyc vector [56] to construct plasmid pGD-P31^NM^-6xMyc, or pGD-P31^O11^-6xMyc, respectively. The cDNA of *P31*^XJ^ or *P31*^OW1^ was synthetized from the Beijing Tsingke Biotech Co., Ltd. and recombined into the pDMC32-FLAG vector [56] to construct plasmid pDMC32-P31^XJ^-FLAG and pDMC32-P31^OW1^-FLAG. To construct plasmid pBN4-P31-FLAG, a fragment containing *P31-FLAG* was amplified from the pDMC32-P31-FLAG [26] and recombined into plasmid pBN4 of deleted-*P31* through inverse PCR method. To obtain plasmid of pGD-P31-GFP, the *P31* cDNA was recombined into the pGD-GFP vector [56]. Plasmids pDMC32-HRD1a-FLAG, pDMC32-HRD1a^2A^-FLAG, pGD-HRD1a-6xMyc, and pGD-HRD1a^2A^-6xMyc have been described previously [36]. For protein purification, the *P31* cDNA was linked to the pDB-His-MBP vector [63] to construct plasmid pDB-His-MBP-P31. The plasmids pGEX-KG-HRD1aC and pGEX-KG-HRD1aC^2A^ have been described in previous studies [36]. For bimolecular fluorescence complementation (BiFC) assays, the pSPYNE-35S vector [64] was inserted with the *P31* cDNA to generate pSPYNE-35S-P31. To generate the plasmid pSPYCE-35S-HRD1a, a fragment of *HRD1a* was engineered into the pSPYCE-35S vector [64]. All primers in this study were listed in S2 Table.

### Plant growth conditions and generation of *NbHRD1*^KO^ transgenic *N*. *benthamiana* plants

Seeds of *B. macrocarpa*, *B. vulgaris*, and *N*. *benthamiana* were vernalized at 4°C for 4 days and grown in soil at 23°C with a 14/10-h light/dark cycle. Through *Agrobacterium*-mediated transformation with pGK01‐*NbHRD1*, the *NbHRD1*^KO^ transgenic *N*. *benthamiana* plants were generated by the leaf disc transformation [65]. Eleven positive *NbHRD1*^KO^ lines were screened independently by Sanger sequencing. Two positive transgenic *NbHRD1*^KO^ lines were used for virus inoculation or ubiquitination assays *in vivo*.

### Virus inoculation assays

The method of BNYVV inoculation has been described in previous studies [66]. Briefly, *Agrobacterium tumefaciens* strain GV3101 harboring pBN1, pBN2, pBN3, and pBN4 were cultivated 12 h at 28°C, collected by a centrifuge, and suspended in suspension buffer (5 mM morpholineethanesulfonic acid, 5 mM MgCl_2_, and 100 mM acetosyringone). pBN1 (OD_600_ = 0.05), pBN2 (OD_600_ = 0.05), pBN3 (OD_600_ = 0.05), and pBN4 (OD_600_ = 0.05) were mixed and infiltrated into leaves of 4-week-old *N*. *benthamiana*, *B. macrocarpa*, or *B. vulgaris*. Systemically infected leaves of *N*. *benthamiana* were harvested for viral RNA and protein analyses at approximately 10 dpi. Systemically infected leaves of *B. macrocarpa* were harvested for viral RNA and protein analyses at 25 dpi. Inoculated leaves of *B. vulgaris* were harvested for viral protein analyses at 15 dpi.

### Protein extraction and immunoblot analysis

Protein extraction and immunoblot analysis were carried out as described previously [67]. Plant total protein was extracted from 0.05 g leaf tissue using 100 μL of 5 × SDS loading buffer containing 0.3% bromophenol blue, 25% glycerol, 8% SDS, 200 mM Tris-HCl (pH 6.8), and 5% DTT. SDS–polyacrylamide gel electrophoresis (SDS–PAGE)-separated proteins were subsequently transferred to nitrocellulose membranes from GE Healthcare company (Buckinghamshire, UK) for detection. Antibodies against TGB1 (1:5,000), coat protein (1:5,000), GFP (1:5,000; Cat. #BE2003, EASYBIO), and P31-FLAG (1:10,000; Cat. #F7425, Sigma-Aldrich) were used for immunoblotting. The antibodies of TGB1 and coat protein were obtained from rabbits in previous studies [15]. The horseradish peroxidase (HRP)-conjugated secondary antibodies (1:5,000; goat anti-rabbit IgG (H&L)-HRP; Cat. #BE0101, EASYBIO) were incubated with nitrocellulose membranes, following primary antibodies incubation. Nitrocellulose membranes were detected using a chemiluminescence (ECL) substrate kit (Cat. #P2300, New Cell & Molecular Biotech). For ERAD inhibitor assay, *N. benthamiana* leaves were co-infiltrated with P31-FLAG and 50 μm kifunensine.

### RNA extraction and reverse transcription-quantitative real-time PCR (RT–qPCR)

RNA extraction and RT–qPCR analysis was performed as previously described [68]. Total RNA was extracted from *B. macrocarpa* or *N*. *benthamiana*, using a TRIzol reagent (Cat. #ET101–01-V2, TransGen Biotech). Total RNA treated with DNase I, M-MLV reverse transcriptase [Cat. #AG11728, Accurate Biotechnology (Hunan)], forward primer for BNYVV *CP*, and oligo (dT_18_) primer were used for cDNA synthesis. Through 2 × M5 HiPer Realtime PCR Super mix (Cat. #MF797–01, Mei5 Biotechnology), target gene fragments were amplified by the ABI QuantStudio 6 Flex system. RT–qPCR results in this study were repeated least three times for ensuring accuracy and reliability.

### Co-immunoprecipitation (Co-IP) assays

Co-IP assays *in vivo* were performed as described previously [69]. Diverse experimental protein combinations were co-expressed in *N*. *benthamiana* leaves. Infiltrated leaves were treated with 100 μM MG132 before 15 h harvest. At 3 dpi, infiltrated-leaves were collected, and plant total proteins were extracted in extraction buffer (50 μM MG132, 25 mM Tris–HCl, pH 7.5, 150 mM NaCl, 1 mM PMSF, 1 mM EDTA, 5 mM DTT, 1% Triton X-100, 5% glycerol, and 2% PVPP) following centrifuge at 5,000 *g* for 20 min. The supernatant was incubated with anti-FLAG (Cat. # FNM-50–2000, Sigma-Aldrich) or anti-MYC (Cat. #MNM-50–2000, LABLEAD) beads at 4°C for 4 h. The precipitates were washed five times with washing buffer (25 mM Tris–HCl, pH 7.5, 10% glycerol, 150 mM NaCl, 1 mM EDTA, and 0.1% Triton X-100), and detected by immunoblot analyses using anti-FLAG (1:10,000; Cat. #F7425, Sigma-Aldrich) or anti-MYC (1:10,000; Cat. #BE5546, Sigma-Aldrich) antibodies.

### *In vitro* Pull-down assays

GST Pull-down assays *in vitro* were carried out as described previously [70]. Briefly, approximately 2 μg purified His-MBP-P31 was incubated with GST-HRD1aC in 500 μL binding buffer (1 mM PMSF, 50 mM Tris–HCl, pH 7.5, 0.2% glycerol, 5 mM DTT, 1% Triton X-100, and 100 mM NaCl) with 10 μL of glutathione-agarose beads from the GE Healthcare company (Buckinghamshire, UK) at 4°C for 4 h, respectively. Then, beads were washed five times with wash buffer (1% Triton X–100, 300 mM NaCl, 50 mM Tris–HCl, pH 7.5, and 0.2% glycerol) and detected by immunoblot using anti-His (1:10,000; Cat. #1029, Sigma-Aldrich) or anti-GST (1:10,000; Cat. #1160, Sigma-Aldrich) antibodies.

### Confocal laser scanning microscopy

HDEL-RFP and P31-GFP were co-expressed in *N*. *benthamiana* leaves. Infiltrated-leaves were treated with 100 μM MG132 at 15 h before confocal observation. At 2 dpi, RFP or GFP fluorescence was observed with a Leica SP8 confocal microscope from the Leica company (Wetzlar, Germany) at 543 nm and 488 nm, respectively.

### Bimolecular fluorescence complementation (BiFC) assays

BiFC assays described earlier were followed [71]. Different expression combinations were infiltrated with *N*. *benthamiana* leaves. Inoculated leaves were treated with 100 μM MG132 at 15 h before confocal observation. At 2 dpi, a Leica SP8 confocal microscope from the Leica company (Wetzlar, Germany) was used to detect the YFP signal.

### Cell fractionation assays

Cell fraction assays were carried out as described previously [36]. Briefly, *N*. *benthamiana* leaves were inoculated with P31-GFP by agroinfiltration. At 2 dpi, inoculated leaves were treated with homogenization buffer (1 mM PMSF, 1 mM DTT, 10 mM KCl, 50 mM Tris–HCl, pH 8.0, 0.3% dextran, 3 mM MgCl_2_, 1 mM EDTA, 0.1% BSA, and 13% sucrose). Two layers of Miracloth with centrifugation at 3,000 *g* to extrude total protein extracts. Then, supernatant was centrifuged at 40,000 *g* to collect the pellet (P30) and soluble (S30) fractions.

### Substrate ubiquitination assays *in vitro*

Substrate ubiquitination assays *in vitro* were carried out as described previously [72]. Reaction mixture (50 μL) contained 800 ng Ub, 400 ng E1 (GI: 136632), 400 ng E2 (UBCh5b), 800 ng GST-HRD1aC, and 800 ng His-MBP-P31. Reacted mixtures at 37°C for 60 min were analyzed through immunoblot using anti-Ub (1:5,000; Cat. #SAB2102632, Sigma-Aldrich) or anti-His (1:10,000; Cat. #1029, Sigma-Aldrich) antibodies.

### *In vivo* ubiquitination assays

Various expression combinations were expressed into 5-week-old *N*. *benthamiana*. Inoculated leaves were treated with 100 μM MG132 at 15 h before harvest. Using the Co-IP assay protocol [73], total proteins were extracted from infiltrated leaves, and the supernatant was incubated with anti-MYC beads or anti-FLAG beads at 4°C for 4 h. After washing six times by wash buffer, the precipitates were analyzed through immunoblot using anti-HA (1:5,000; Cat. #0906–1, HUABIO), anti-MYC (1:10,000; Cat. #BE5546, Sigma-Aldrich), or anti-FLAG (1:10,000; Cat. #F7425, Sigma-Aldrich) antibodies.

### Cell-free protein degradation assays

Cell-free protein degradation assays have been described previously [36]. Briefly, 0.2 g *N*. *benthamiana* leaf tissues were ground in 500 μL native buffer (1 mM MgCl_2_, 5 mM DTT, 10 mM EDTA, pH 7.5, 0.5 M sucrose, and 50 mM Tris–HCl, pH 7.5) following centrifuge at 10,000 *g* for 20 min. The supernatant was mixed with His-MBP-P31 and reacted at room temperature for different times.

For 26S proteasome-mediated protein degradation assays, 50 μM CHX, 20 mM ATP, and 50 μM MG132 were added into the leaf protein extracts with His-MBP-P31. Mixtures were incubated at room temperature and collected for 0, 120, and 240 min followed by immunoblot with anti-His antibodies.

For analyzing the influence of HRD1a-FLAG or HRD1a^2A^-FLAG on the P31 protein levels, HRD1a-FLAG or HRD1a^2A^-FLAG was expressed into *N*. *benthamiana* leaves by agroinfiltration, respectively. At 2 dpi, leaf tissues were ground in native buffer, and mixed with His-MBP-P31, CHX, and ATP. Mixtures were reacted at room temperature and collected for 0, 60, and 120 min followed by immunoblot with anti-His antibodies and anti-FLAG antibodies.

### Split-TurboID-based proximity labeling assay and mass spectrometry (LC-MS/MS) analysis

Split-TurboID-based proximity labeling assays were carried out as described previously [63]. P31^NM^-TurboID-GFP or TurboID-GFP was expressed into *N*. *benthamiana* leaves. At 40 hpi, infiltrated leaves were treated with 100 μm biotin and harvested at 48 hpi. Biotinylated proteins were incubated with streptavidin beads (Cat. #NO. 65001, Invitrogen), followed by on-bead trypsin digestion. Then LC-MS/MS analyses were carried out to identify biotinylated proteins. The mass spectrometry proteomics data have been deposited to the ProteomeXchange Consortium (https://proteomecentral.proteomexchange.org) via the iProX partner repository [74] with the dataset identifier PXD071299.

### Phylogenetic analysis

The aa sequences of P31 from different RNA4 isolates were obtained from the NCBI website (https://www.ncbi.nlm.nih.gov/). Phylogenetic analyses of P31 were performed through the ClustalW method in MEGAX. With 1,000 bootstrap replicates, a maximum likelihood tree was established. The sequences used for constructing phylogenetic analyses in the article are as follows: the amino acid sequences of P31 of BNYVV RNA4 from Chinese BT-1 isolate (NP_612622.1), France FC isolate (AB563128.1), Chinese CY3 isolate (AB563117.1), France FB isolate (AB563131.1), Germany GW isolate (AB563133.1), Germany GM isolate (AB563136.1), England UK-FF isolate (ABD97955.1), and BSBMV P32 (YP_009513208.1). The sequences of Chinese NM isolate (XXM59340), Chinese XJ isolate (AKI85790.1), Japanese O11 isolate (BAJ23818.1), and Germany OW1 isolate (EU864119.1) are exhitbited in S2 Fig.

### Quantification in immunoblot and statistical analysis

The ImageJ software was used to measure each band intensity in immunoblot analyses for quantification [68]. Statistical analysis of RT–qPCR is determined by the Student’s t-test (****P < 0.0001, ***P < 0.001, **P < 0.01, and *P < 0.05) or the Dunnett’s multiple comparison test (different letters suggest a significant discrepancy at *P* < 0.05) [75–77]. All values in RT–qPCR are presented as means ± SD.

### Accession numbers

Accession numbers for related genes in the article are as follows: The *P31* gene of BNYVV RNA4 from Chinese NM isolate (XXM59340), Chinese XJ isolate (AKI85790.1), Chinese BT-1 isolate (NP_612622.1), Japanese O11 isolate (BAJ23818.1), France FC isolate (AB563128.1), Chinese CY3 isolate (AB563117.1), France FB isolate (AB563131.1), Germany GW isolate (AB563133.1), Germany GM isolate (AB563136.1), Germany OW1 isolate (EU864119.1), England UK-FF isolate (ABD97955.1), and BSBMV *P32* (YP_009513208.1) can be obtained from the NCBI website. In addition, other genes discussed in the article: *HRD1a* (Nbe06g19750.1) or *HRD1b* (Nbe05g21720.1) can be accessed on the *N*. *benthamiana* and *tabacum Omics* website (http://lifenglab.hzau.edu.cn/Nicomics/) [78].

## Supporting information

S1 Table The related information of P31 proteins in different countries. (DOCX)

S2 Table List of primers in this study. (DOCX)

S1 Fig (A) Schematic representation of pBN1, pBN2, pBN2-GFP, pBN3, and pBN4-FLAG plasmids for expression of BNYVV RNA1, RNA2, RNA2-GFP, RNA3, and RNA4. RdRP, RNA-dependent RNA polymerase; RTD, read-through protein; TGB, triple gene block, including TGB1, TGB2, and TGB3. (B) RT–qPCR anylsis of viral genomic RNA levels in the samples in **Fig 1E**. The viral *CP* gene was used as an indicator of viral RNA levels. *EF1α* was used as an internal control. Error bars indicate means ± SD of three biological repeats. Letters indicate significant differences (ANOVA, *P* < 0.05).
(TIF)

S2 Fig The co-conserved nucleotide sequence was indicated by black color. (TIF)

S3 Fig (A) Schematic representation of the constructs used for identification of P31^NM^ interacted proteins. (B) Diagram of experimental design. *Agrobacterium tumefaciens* harboring the P31^NM^ -TurboID-GFP or the TurboID-GFP construct was inoculated into *Nicotiana benthamiana* leaves. At 40 hours post-infiltration (hpi), 200 μm biotin was infiltrated into the same leaves. The leaf sample was harvested after 8 hours. Biotinylated proteins were enriched with streptavidin beads followed by on-bead trypsin digestion. Then the LC-MS/MS analysis was performed to identify the biotinylated proteins. Each experiment was carried out with three separate biological replicates (n = 3 plants for each replicate). (C) Immunoblot analysis of protein expression and biotinylation in panel (B).
(TIF)

S4 Fig Immunoblotting analysis of protein expression in Fig 4D probed with anti-HA and anti-Myc antibodies. RbcL served as loading control. (TIF)

S5 Fig (A) Immunoblot analyzing the effect of HRD1a on P31^O11^ ubiquitination *in vivo*. *N. benthamiana* leaves co-expressing HRD1a-Myc with P31^O11^-FLAG or mCherry-FLAG. The mCherry-FLAG served as a negative control. Total protein extracts were immunoprecipitated with anti-FLAG beads, followed by immunoblot analysis with the indicated antibodies. (B) Immunoblot analyzing the effect of HRD1a on P31^NM^, P31^NM-R147K^, P31^O11^, and P31^O11-K147R^ ubiquitination *in vivo*. *N. benthamiana* leaves co-expressing P31-FLAG with HRD1a-Myc or HRD1a^Cys307Ala/His312Ala^ (HRD1a^2A^)-Myc. protein extracts were immunoprecipitated with anti-Myc beads, followed by immunoblot analysis with the indicated antibodies. The red asterisk represents weight chains.
(TIF)

S6 Fig The red box indicated guide RNA sequence was chosen from the nt 108–127 region downstream of the *HRD1a* start codon, which was a conserved region in *HRD1a* and *HRD1b.* (TIF)

S7 Fig (A–B) Sequencing analysis of the insertion or deletion mutation regions of *NbHRD1a* and *NbHRD1b* in *NbHRD1*^KO^ transgenic plants. (C) Representative images of 2-week-old NT and two *NbHRD1*^KO^ lines (#1, #2). Scale bars, 2.5 cm. (D) Representative images of 4-week-old NT and two *NbHRD1*^KO^ lines (#1, #2). Scale bars, 5 cm. (E) Above ground fresh weight of plants for panel **(D)**. Error bar represents ±SD of 9 plants. Ns, not significance. (Student’s t‐test).
(TIF)

S1 Raw Data Values to build graphs and intensity ratio of western blot bands in all figures. (XLSX)
