# Supplementary material for: Natural genetic variation of a single amino acid in beet necrotic yellow vein virus P31 protein modulates evasion of plant ubiquitination-mediated antiviral immunity
Source: PLoS Pathog. 2026 Jan 2;22(1):e1013840. doi: 10.1371/journal.ppat.1013840 (PMC12782415; doi:10.1371/journal.ppat.1013840)
Supplement: S1 Table — (DOCX) [file ppat.1013840.s001.docx]

S1 **Table.** **The related information of P31 proteins in different countries.**

| **Accession number** | **Country** | **Isolate** | **AA147** |
| --- | --- | --- | --- |
| UZT75598 | Turkey | ANK-617 | k |
| UZT75597 | Turkey | KAS-281 | k |
| UZT75596 | Turkey | CNK-150 | k |
| UZT75595 | Turkey | KYS-524 | k |
| UZT75594 | Turkey | BRS-148 | k |
| UZT75593 | Turkey | EDR-125 | k |
| UZT75592 | Turkey | SMS-61 | k |
| UZT75591 | Turkey | ELZ-44 | k |
| UZT75590 | Turkey | GZP-27 | k |
| UZT75589 | Turkey | ERC-52 | k |
| UZT75588 | Turkey | IGR-9 | k |
| UZT75587 | Turkey | IGR-6 | k |
| QKV49885 | USA | S6 | k |
| QKV49883 | USA | S5 | k |
| QKV49881 | USA | S4 | k |
| QKV49879 | USA | S3 | k |
| QKV49877 | USA | S2 | k |
| QKV49875 | USA | S1 | k |
| BAJ23863 | USA | USM | k |
| BAJ23862 | USA | USTH | k |
| BAJ23861 | Spain | SPC | k |
| BAJ23860 | Hungary | HUT4 | k |
| BAJ23859 | Hungary | HUM1 | k |
| BAJ23858 | Slovakia | SLP2 | k |
| BAJ23857 | Slovakia | SLN1 | k |
| BAJ23856 | Germany | GM | k |
| BAJ23855 | Germany | GA | k |
| BAJ23854 | Germany | GI | k |
| BAJ23853 | Germany | GW | k |
| BAJ23852 | France | FAH | k |
| BAJ23851 | France | FB | k |
| BAJ23850 | France | FAO | k |
| BAJ23849 | Italy | IF3 | k |
| BAJ23848 | France | FC | k |
| BAJ23847 | France | FP | k |
| BAJ23846 | Italy | IP7 | k |
| BAJ23845 | Italy | IF6 | k |
| BAJ23844 | Italy | IV4 | k |
| BAJ23843 | Italy | IV1 | k |
| BAJ23842 | China | CHa | r |
| BAJ23841 | China | CX6 | r |
| BAJ23840 | China | CX5 | r |
| BAJ23839 | China | CX3 | r |
| BAJ23838 | China | CX1 | k |
| BAJ23837 | China | CY3 | k |
| BAJ23836 | China | CY1 | r |
| BAJ23835 | China | CW11 | r |
| BAJ23834 | China | CW1 | r |
| BAJ23833 | China | CH7 | r |
| BAJ23832 | China | CH5 | r |
| BAJ23831 | China | CH4 | r |
| BAJ23830 | China | CH3 | r |
| BAJ23829 | China | CH2 | r |
| BAJ23828 | Japan | H45 | r |
| BAJ23827 | Japan | T16 | r |
| BAJ23826 | Japan | T41 | r |
| BAJ23825 | Japan | S44 | r |
| BAJ23824 | Japan | S12 | r |
| BAJ23823 | Japan | S42 | r |
| BAJ23822 | Japan | N64 | k |
| BAJ23821 | Japan | K53 | k |
| BAJ23820 | Japan | M87 | k |
| BAJ23819 | Japan | SH1 | r |
| BAJ23818 | Japan | O11 | k |
| BAJ23817 | Japan | K80 | k |
| BAJ23816 | Japan | R83 | k |
| BAJ23815 | Japan | S113 | k |
| BAJ23814 | Japan | S110 | k |
| BAJ23813 | Japan | T101 | k |
| ABG66422 | France | Pithiviers | k |
| CAR66232 | Iran | No | k |
| UZZ47394 | Germany | DSMZ PV-0990 | k |
| XCG44736 | Kazakhstan | Kz2-58 | k |
| XCG44735 | Kazakhstan | Kz2-51 | k |
| XCG44734 | Kazakhstan | Kz2-41 | k |
| XCG44733 | Kazakhstan | Kz2-26 | k |
| XCG44732 | Kazakhstan | Kz2-10 | k |
| XCG44731 | Kazakhstan | Kz2-05 | k |
| XCG44730 | Kazakhstan | Kz2-01 | k |
| XCG44729 | Kazakhstan | Kz1-8 | k |
| XCG44728 | Kazakhstan | Kz1-49 | k |
| XCG44727 | Kazakhstan | Kz1-3 | k |
| XCG44726 | Kazakhstan | Kz1-28 | k |
| XCG44725 | Kazakhstan | Kz1-16 | k |
| XCG44724 | Kazakhstan | Kz1-10 | k |
| XCG44723 | Kazakhstan | Kz1-32 | k |
| UWY64071 | Turkey | A | k |
| UWY64070 | Turkey | A/B | k |
| UWY64069 | Turkey | A/B | k |
| UWY64068 | Turkey | B | k |
| UWY64067 | Turkey | B | k |
| UWY64066 | Turkey | B | k |
| UWY64065 | Turkey | A/B | k |
| UWY64064 | Turkey | B | k |
| UWY64063 | Turkey | B | k |
| UWY64062 | Turkey | B | k |
| UWY64061 | Turkey | B | k |
| UWY64060 | Turkey | A | k |
| UWY64059 | Turkey | A/B | k |
| AAU20336 | Iran | BNQ1 | t |
| ABA41486 | Iran | M-28 | t |
| CAO91857 | Iran | IR-GR | k |
| Q65670 | No | No | r |
| ACJ64686 | Germany | OW1 | k |
| ABD97955 | United Kingdom | No | r |
| ABD97954 | United Kingdom | No | k |
| ABS19952 | China | Chan2 | r |
| ABS19951 | China | Chan1 | r |
| ABS19950 | China | Wu2 | r |
| ABS19949 | China | Wu1 | r |
| ABS19948 | China | Bao | k |
| ABS19947 | China | Hoh3 | r |
| ABS19946 | China | Hoh1 | r |
| ABS19945 | China | Har4 | k |
| AKI85352 | China | IMHHP31-9 | r |
| AKI85351 | China | IMHHP31-8 | r |
| AKI85350 | China | IMHHP31-7 | r |
| AKI85349 | China | IMHHP31-6 | r |
| AKI85348 | China | IMHHP31-5 | r |
| AKI85347 | China | IMHHP31-4 | r |
| AKI85346 | China | IMHHP31-3 | r |
| AKI85345 | China | IMHHP31-2 | r |
| AKI85344 | China | IMHHP31-1 | r |
| AKI85807 | China | JJCP31-6 | r |
| AKI85806 | China | JJCP31-5 | r |
| AKI85805 | China | JJCP31-4 | r |
| AKI85804 | China | JJCP31-3 | r |
| AKI85803 | China | JJCP31-2 | r |
| AKI85802 | China | JJCP31-1 | r |
| AKI85801 | China | CBCEP31-12 | r |
| AKI85800 | China | CBCEP31-11 | r |
| AKI85799 | China | CBCEP31-10 | r |
| AKI85798 | China | CBCEP31-9 | r |
| AKI85797 | China | CBCEP31-8 | r |
| AKI85796 | China | CBCEP31-7 | r |
| AKI85795 | China | CBCEP31-6 | r |
| AKI85794 | China | CBCEP31-5 | r |
| AKI85793 | China | CBCEP31-4 | r |
| AKI85792 | China | CBCEP31-3 | r |
| AKI85791 | China | CBCEP31-2 | r |
| AKI85790 | China | CBCEP31-1 | r |
| AKI85789 | China | LYP31-11 | r |
| AKI85788 | China | LYP31-10 | r |
| AKI85787 | China | LYP31-9 | r |
| AKI85786 | China | LYP31-8 | r |
| AKI85785 | China | LYP31-7 | r |
| AKI85784 | China | LYP31-6 | r |
| AKI85783 | China | LYP31-5 | r |
| AKI85782 | China | LYP31-4 | r |
| AKI85781 | China | LYP31-3 | r |
| AKI85780 | China | LYP31-2 | r |
| AKI85779 | China | LYP31-1 | r |
| AKI85778 | China | YAP31-8 | r |
| AKI85777 | China | YAP31-7 | r |
| AKI85776 | China | YAP31-6 | r |
| AKI85775 | China | YAP31-5 | r |
| AKI85774 | China | YAP31-4 | r |
| AKI85773 | China | YAP31-3 | r |
| AKI85772 | China | YAP31-2 | r |
| AKI85771 | China | YAP31-1 | r |
| AKI85770 | China | HLP31-10 | r |
| AKI85769 | China | HLP31-9 | r |
| AKI85768 | China | HLP31-8 | r |
| AKI85767 | China | HLP31-7 | r |
| AKI85766 | China | HLP31-6 | r |
| AKI85765 | China | HLP31-5 | r |
| AKI85764 | China | HLP31-4 | r |
| AKI85763 | China | HLP31-3 | r |
| AKI85762 | China | HLP31-2 | r |
| AKI85761 | China | HLP31-1 | r |
| AKI85760 | China | KYP31-9 | r |
| AKI85759 | China | KYP31-8 | r |
| AKI85758 | China | KYP31-7 | r |
| AKI85757 | China | KYP31-6 | r |
| AKI85756 | China | KYP31-5 | r |
| AKI85755 | China | KYP31-4 | r |
| AKI85754 | China | KYP31-3 | r |
| AKI85753 | China | KYP31-2 | r |
| AKI85752 | China | KYP31-1 | r |
| AKI85751 | China | HXL291P31-3 | r |
| AKI85750 | China | HXL291P31-2 | r |
| AKI85749 | China | HXL291P31-1 | r |
| AKI85748 | China | YJZP31-9 | r |
| AKI85747 | China | YJZP31-8 | r |
| AKI85746 | China | YJZP31-7 | r |
| AKI85745 | China | YJZP31-6 | r |
| AKI85744 | China | YJZP31-5 | r |
| AKI85743 | China | YJZP31-4 | r |
| AKI85742 | China | YJZP31-3 | r |
| AKI85741 | China | YJZP31-2 | r |
| AKI85740 | China | YJZP31-1 | r |
| AKI85739 | China | GZCP31-8 | r |
| AKI85738 | China | GZCP31-7 | r |
| AKI85737 | China | GZCP31-6 | r |
| AKI85736 | China | GZCP31-5 | r |
| AKI85735 | China | GZCP31-4 | r |
| AKI85734 | China | GZCP31-3 | r |
| AKI85733 | China | GZCP31-2 | r |
| AKI85732 | China | GZCP31-1 | r |
| AKI85731 | China | ZYP31-6 | r |
| AKI85730 | China | ZYP31-5 | r |
| AKI85729 | China | ZYP31-4 | r |
| AKI85728 | China | ZYP31-3 | r |
| AKI85727 | China | ZYP31-2 | r |
| AKI85726 | China | ZYP31-9 | r |
| AKI85725 | China | ZYP31-8 | r |
| AKI85724 | China | ZYP31-7 | r |
| AKI85723 | China | ZYP31-1 | r |
| AKI85722 | China | DHXP31-14 | r |
| AKI85721 | China | DHXP31-13 | r |
| AKI85720 | China | DHXP31-12 | r |
| AKI85719 | China | DHXP31-11 | r |
| AKI85718 | China | DHXP31-10 | r |
| AKI85717 | China | DHXP31-9 | r |
| AKI85716 | China | DHXP31-8 | r |
| AKI85715 | China | DHXP31-7 | r |
| AKI85714 | China | DHXP31-6 | r |
| AKI85713 | China | DHXP31-5 | r |
| AKI85712 | China | DHXP31-4 | r |
| AKI85711 | China | DHXP31-3 | r |
| AKI85710 | China | DHXP31-2 | r |
| AKI85709 | China | DHXP31-1 | r |
| AKI85708 | China | SD18P31-12 | r |
| AKI85707 | China | SD18P31-11 | r |
| AKI85706 | China | SD18P31-10 | r |
| AKI85705 | China | SD18P31-9 | r |
| AKI85704 | China | SD18P31-8 | r |
| AKI85703 | China | SD18P31-7 | r |
| AKI85702 | China | SD18P31-6 | r |
| AKI85701 | China | SD18P31-5 | r |
| AKI85700 | China | SD18P31-4 | r |
| AKI85699 | China | SD18P31-3 | r |
| AKI85698 | China | SD18P31-2 | r |
| AKI85697 | China | SD18P31-1 | r |
| AKI85696 | China | BTP31-16 | r |
| AKI85695 | China | BTP31-15 | r |
| AKI85694 | China | BTP31-14 | r |
| AKI85693 | China | BTP31-13 | r |
| AKI85692 | China | BTP31-12 | r |
| AKI85691 | China | BTP31-11 | r |
| AKI85690 | China | BTP31-10 | r |
| AKI85689 | China | BTP31-9 | r |
| AKI85688 | China | BTP31-8 | r |
| AKI85687 | China | BTP31-7 | r |
| AKI85686 | China | BTP31-6 | r |
| AKI85685 | China | BTP31-5 | r |
| AKI85684 | China | BTP31-4 | r |
| AKI85683 | China | BTP31-3 | r |
| AKI85682 | China | BTP31-2 | r |
| AKI85681 | China | BTP31-1 | r |
| AKI85452 | China | SDTCP31-13 | r |
| AKI85451 | China | SDTCP31-12 | r |
| AKI85450 | China | SDTCP31-11 | r |
| AKI85449 | China | SDTCP31-10 | r |
| AKI85448 | China | SDTCP31-9 | r |
| AKI85447 | China | SDTCP31-8 | r |
| AKI85446 | China | SDTCP31-7 | r |
| AKI85445 | China | SDTCP31-6 | r |
| AKI85444 | China | SDTCP31-5 | r |
| AKI85443 | China | SDTCP31-4 | r |
| AKI85442 | China | SDTCP31-3 | r |
| AKI85441 | China | SDTCP31-2 | r |
| AKI85440 | China | SDTCP31-1 | r |
| AIE17036 | No | No | t |
