# Supplementary material for: Natural genetic variation of a single amino acid in beet necrotic yellow vein virus P31 protein modulates evasion of plant ubiquitination-mediated antiviral immunity
Source: PLoS Pathog. 2026 Jan 2;22(1):e1013840. doi: 10.1371/journal.ppat.1013840 (PMC12782415; doi:10.1371/journal.ppat.1013840)
Supplement: S2 Table — (DOCX) [file ppat.1013840.s002.docx]

| **Primer name** | **Sequence (5'-3')** | **Notes** |
| --- | --- | --- |
| pGD-P31-Myc-F | TCTCTCTACAAGATCTCGAG ATGGCTGATG GAGAGATATG | pGD-P31-Myc |
| pGD-P31-Myc-R | CTCCGCCGGA TCCCGGGCCC ATCGTGATAA AAGACAAACC |  |
| pBN4-ΔP31-F | ATGG AGAGATATGT CGGTGTCAGG | pBN4-ΔP31 |
| pBN4-ΔP31-R | CAGCCATAAA TGTTACTAAT CAGATA |  |
| pGD-P31-GFP-F | CTCTCTCTACAAGATCTCGAG ATGGCTGATG GAGAGATATG | pGD-P31-GFP |
| pGD-P31-GFP-R | CTTCTCCTTTACTCATGGGCCCATCGTGATAA AAGACAAACC |  |
| pDB-His-MBP-P31-F | TACTTCCAGGGCCATATG ATGGCTGATG GAGAGATATG | pDB-His-MBP-P31 |
| pDB-His-MBP-P31-R | TGGTGGTGGT GGTGCTCGAGTATCGTGATAA AAGACAAACC |  |
| pSPYNE-35S-P31-F | CGCCACTAGTGGATCC ATGGCTGATG GAGAGATATG | pSPYNE-35S-P31 |
| pSPYNE-35S-P31-R | TACTATCGATGGATCC ATCGTGATAA AAGACAAACC |  |
| pSPYCE-35S-HD1-F | CGCCACTAGTGGATCC ATGATGAGGT TACAAACTTA | pSPYCE-35S-HRD1 |
| pSPYCE-35S-HD1-R | TACTATCGATGGATCC CATTTCTGCA CCTTCGGTTT |  |
| BN2-qRT-F | TTACCATG GACACCTGTTCAAGG | RT–qPCR |
| BN2-qRT-R | AGGATATAA TAGTGCCCGCTTCG |  |
| BmActin-qRT-F | CAGGTATTGTGCTTGACTCT |  |
| BmActin-qRT-R | AACATGTAACCTCTTTCGGT |  |
| NbEF1α-qRT-F | GATTAATG AGCCCAA GAGGCC |  |
| NbEF1α-qRT-R | AGTTTC CACAC GACCAACAGG |  |

S2 Table. List of primers in this study.
