# Supplementary figures and images for: Natural genetic variation of a single amino acid in beet necrotic yellow vein virus P31 protein modulates evasion of plant ubiquitination-mediated antiviral immunity
Source: PLoS Pathog. 2026 Jan 2;22(1):e1013840. doi: 10.1371/journal.ppat.1013840 (PMC12782415; doi:10.1371/journal.ppat.1013840)

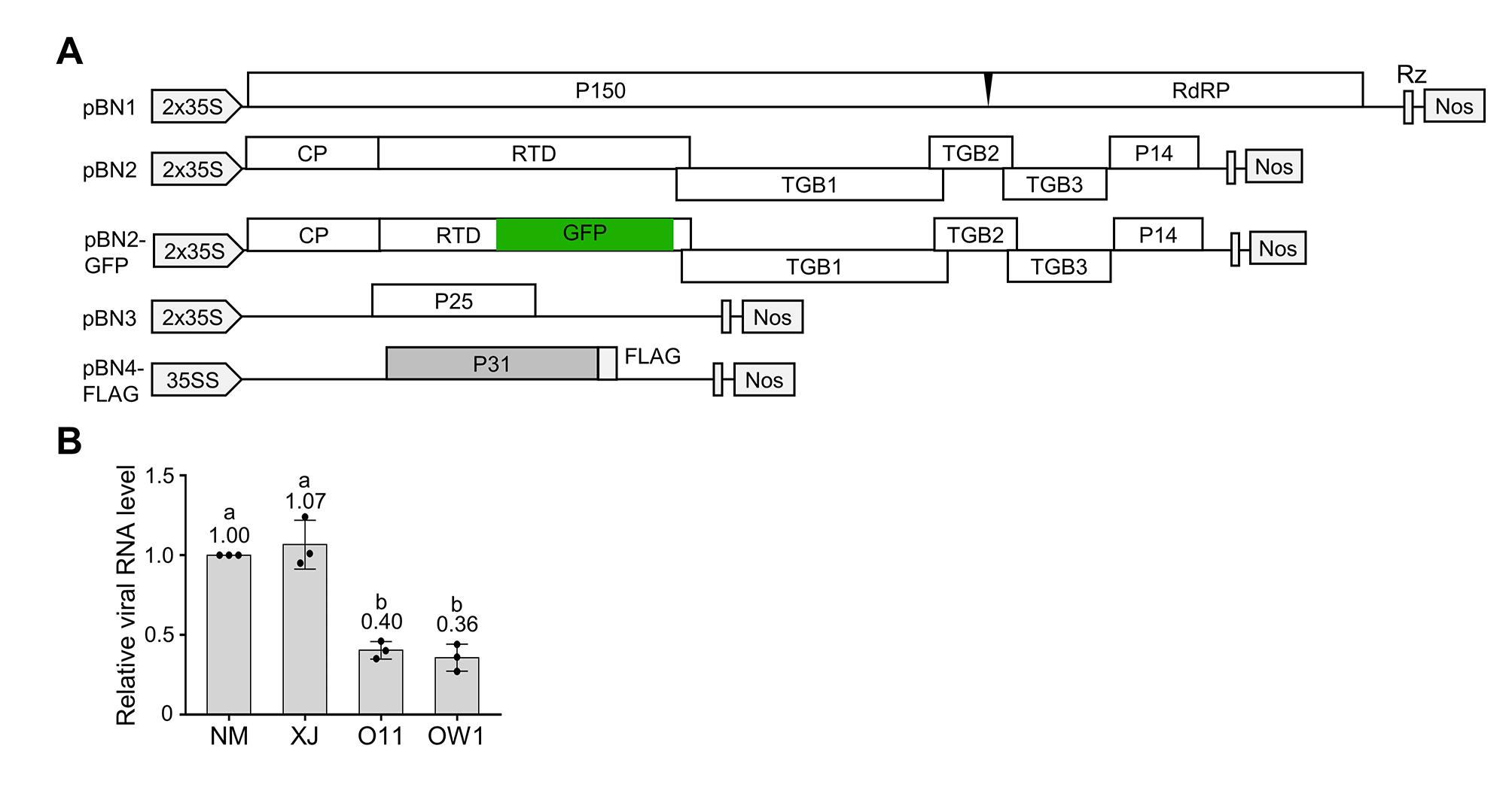

Supplement: S1 Fig — RdRP, RNA-dependent RNA polymerase; RTD, read-through protein; TGB, triple gene block, including TGB1, TGB2, and TGB3. (B) RT–qPCR anylsis of viral genomic RNA levels in the samples in Fig 1E. The viral CP gene was used as an indicator of viral RNA levels. EF1α was used as an internal control. Error bars indicate means ± SD of three biological repeats. Letters indicate significant differences (ANOVA, P < 0.05). (TIF) [file ppat.1013840.s003.tif]

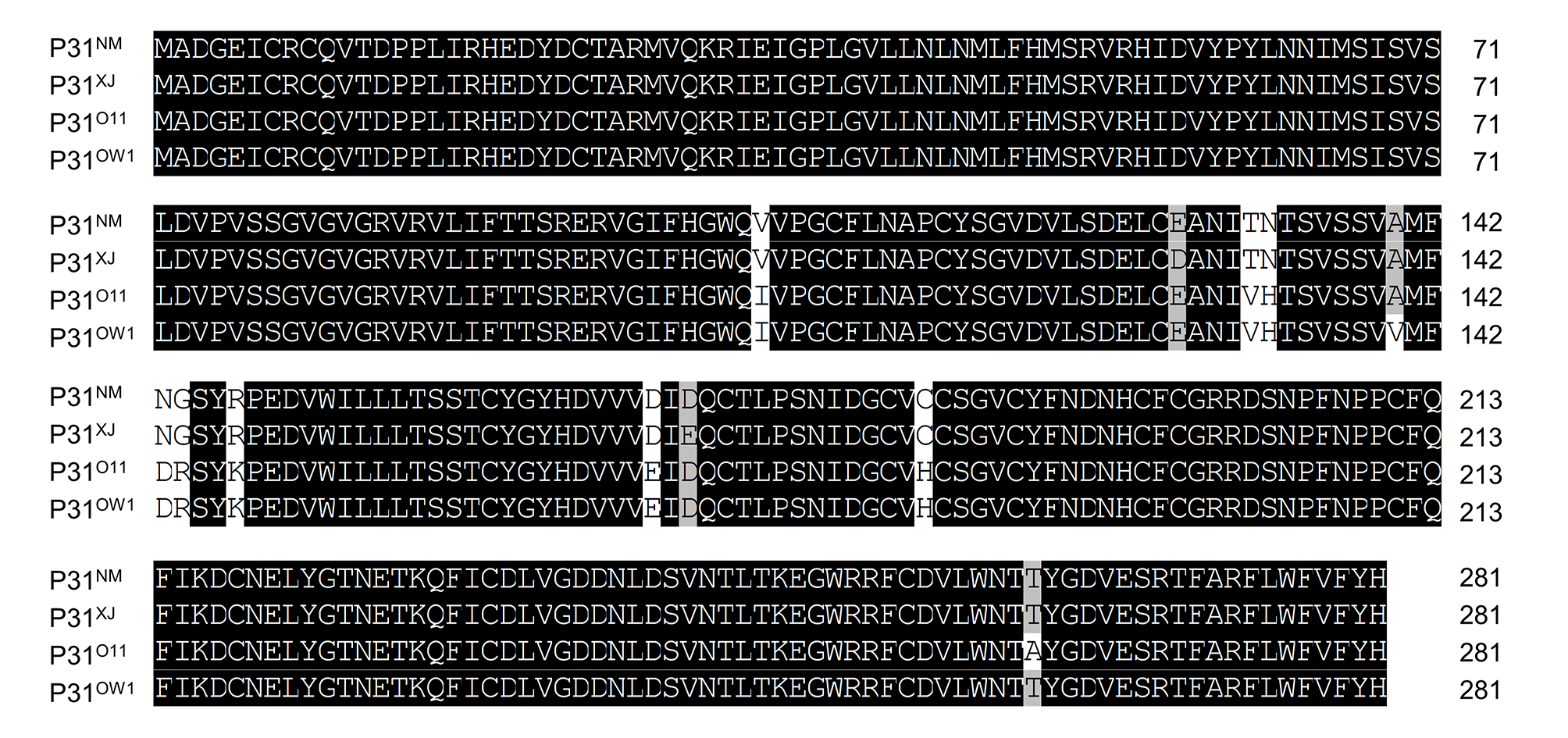

Supplement: S2 Fig — (TIF) [file ppat.1013840.s004.tif]

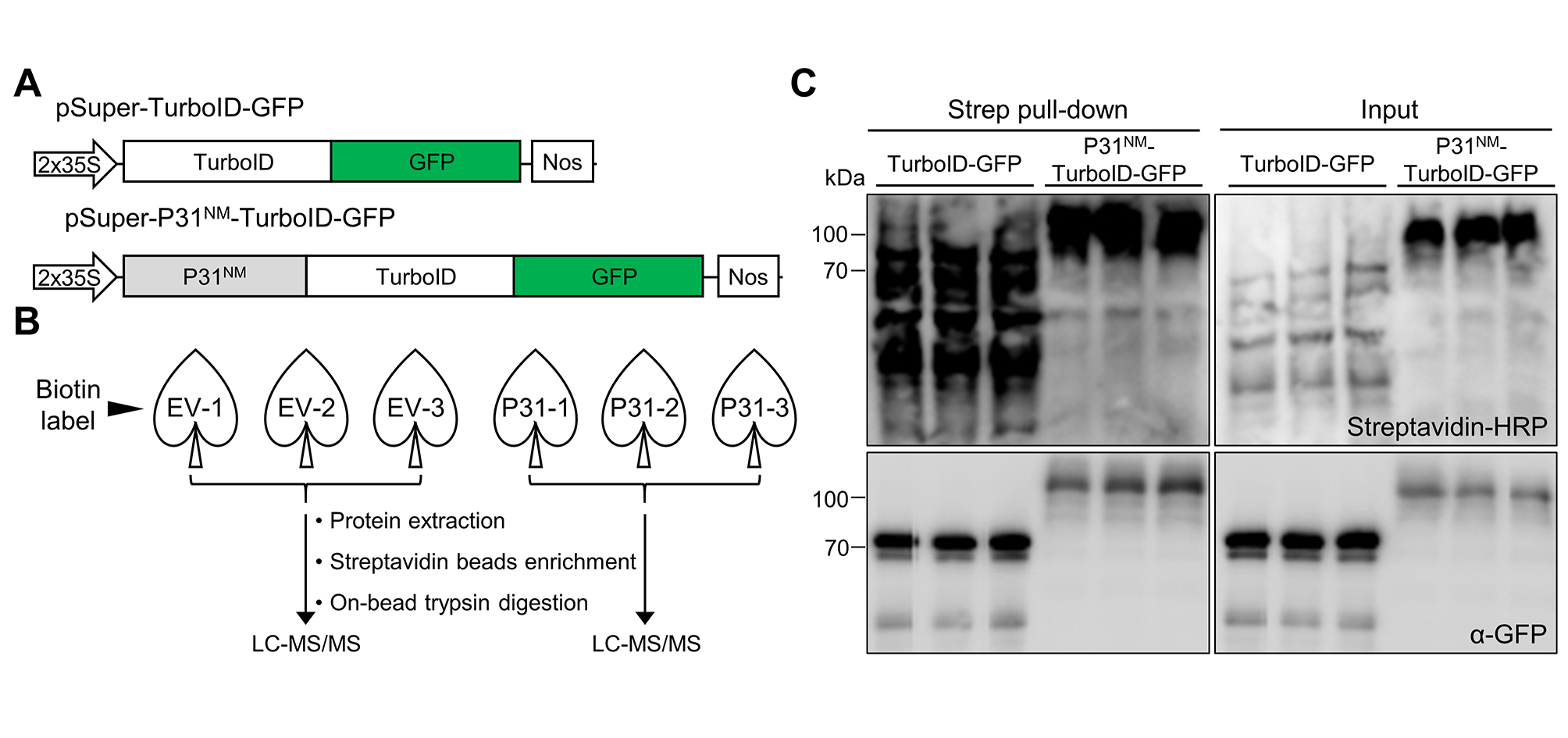

Supplement: S3 Fig — (A) Schematic representation of the constructs used for identification of P31NM interacted proteins. (B) Diagram of experimental design. Agrobacterium tumefaciens harboring the P31NM -TurboID-GFP or the TurboID-GFP construct was inoculated into Nicotiana benthamiana leaves. At 40 hours post-infiltration (hpi), 200 μm biotin was infiltrated into the same leaves. The leaf sample was harvested after 8 hours. Biotinylated proteins were enriched with streptavidin beads followed by on-bead trypsin digestion. Then the LC-MS/MS analysis was performed to identify the biotinylated proteins. Each experiment was carried out with three separate biological replicates (n = 3 plants for each replicate). (C) Immunoblot analysis of protein expression and biotinylation in panel (B). (TIF) [file ppat.1013840.s005.tif]

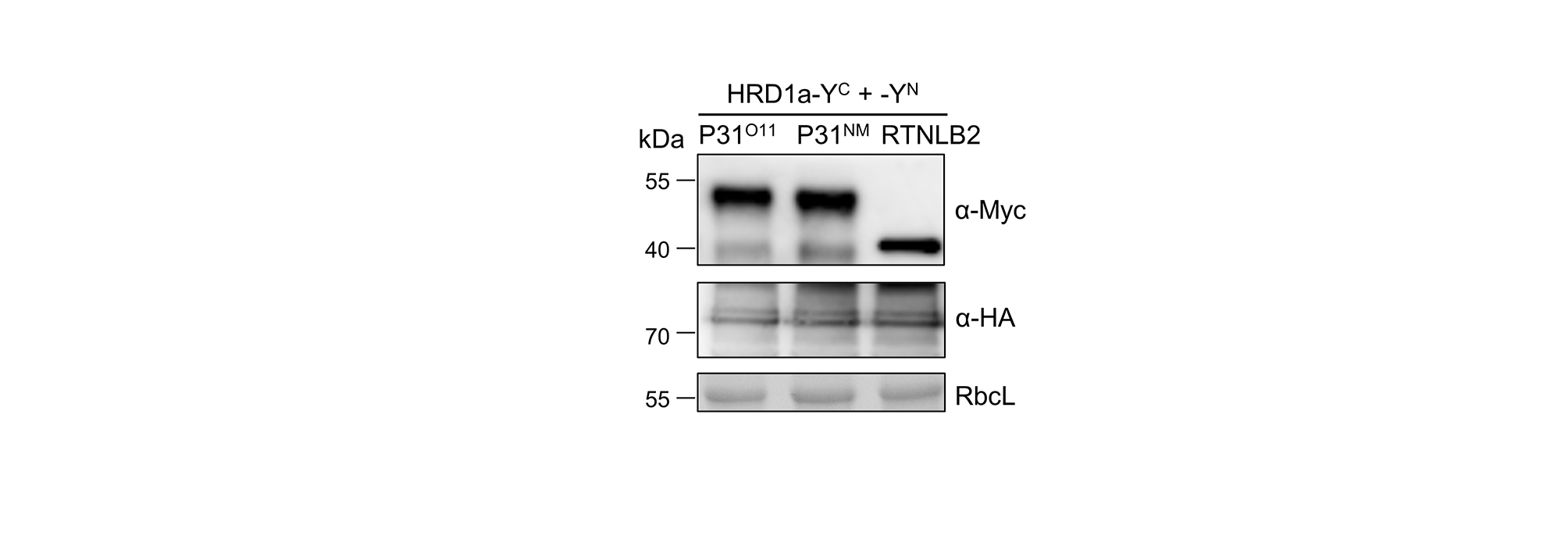

Supplement: S4 Fig — (TIF) [file ppat.1013840.s006.tif]

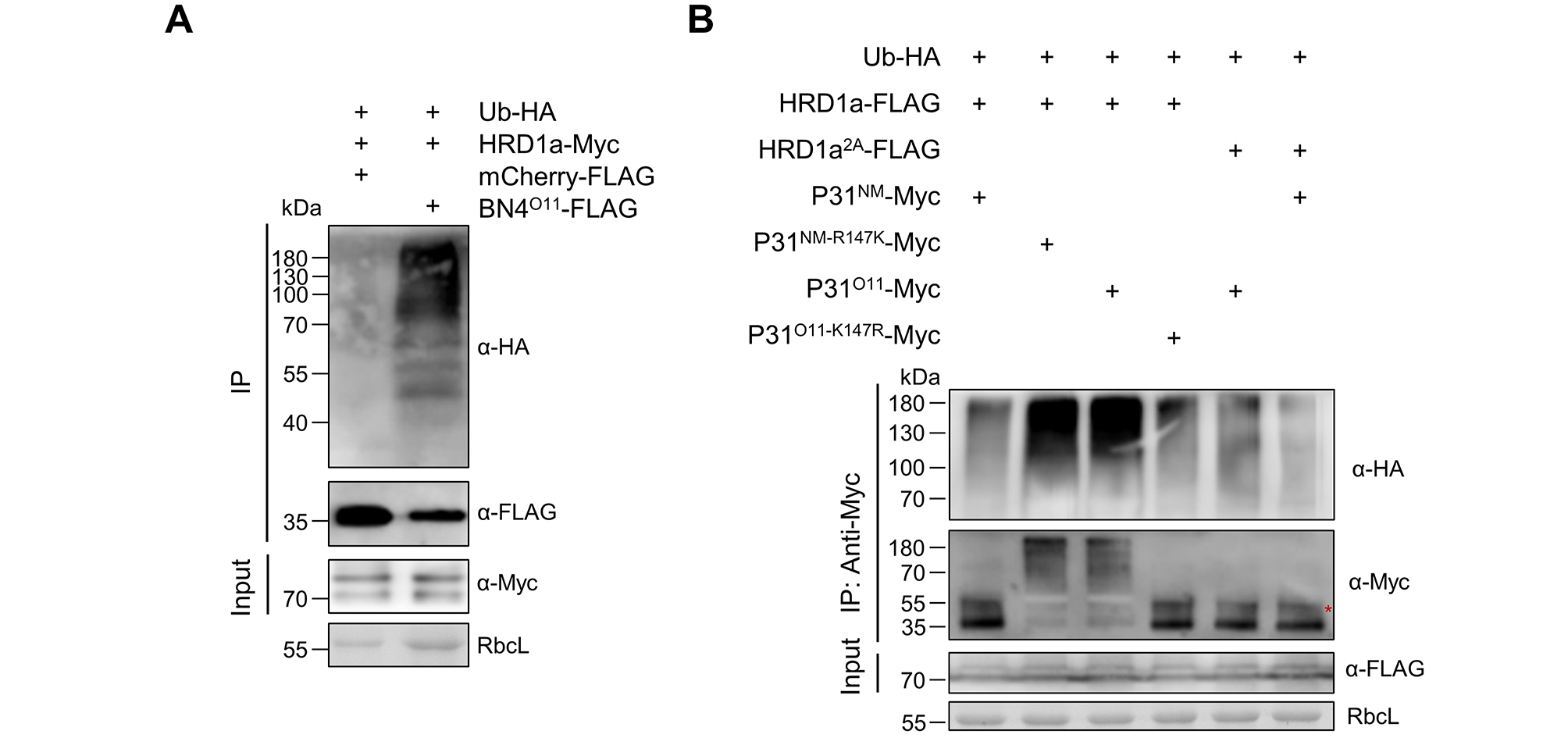

Supplement: S5 Fig — The mCherry-FLAG served as a negative control. Total protein extracts were immunoprecipitated with anti-FLAG beads, followed by immunoblot analysis with the indicated antibodies. (B) Immunoblot analyzing the effect of HRD1a on P31NM, P31NM-R147K, P31O11, and P31O11-K147R ubiquitination in vivo. N. benthamiana leaves co-expressing P31-FLAG with HRD1a-Myc or HRD1aCys307Ala/His312Ala (HRD1a2A)-Myc. protein extracts were immunoprecipitated with anti-Myc beads, followed by immunoblot analysis with the indicated antibodies. The red asterisk represents weight chains. (TIF) [file ppat.1013840.s007.tif]

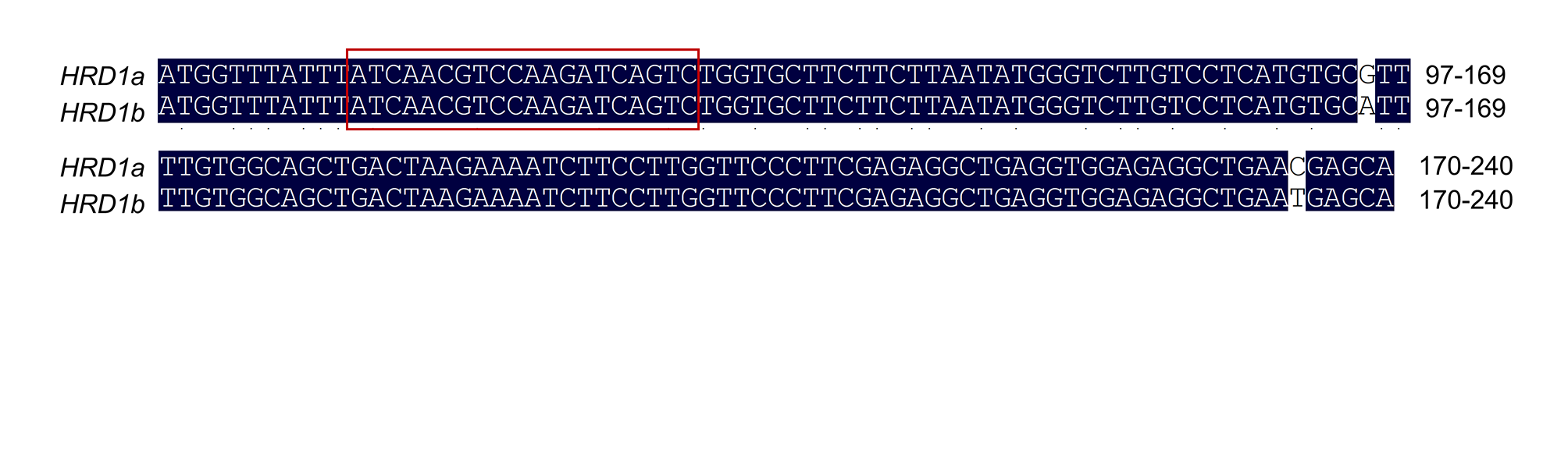

Supplement: S6 Fig — (TIF) [file ppat.1013840.s008.tif]

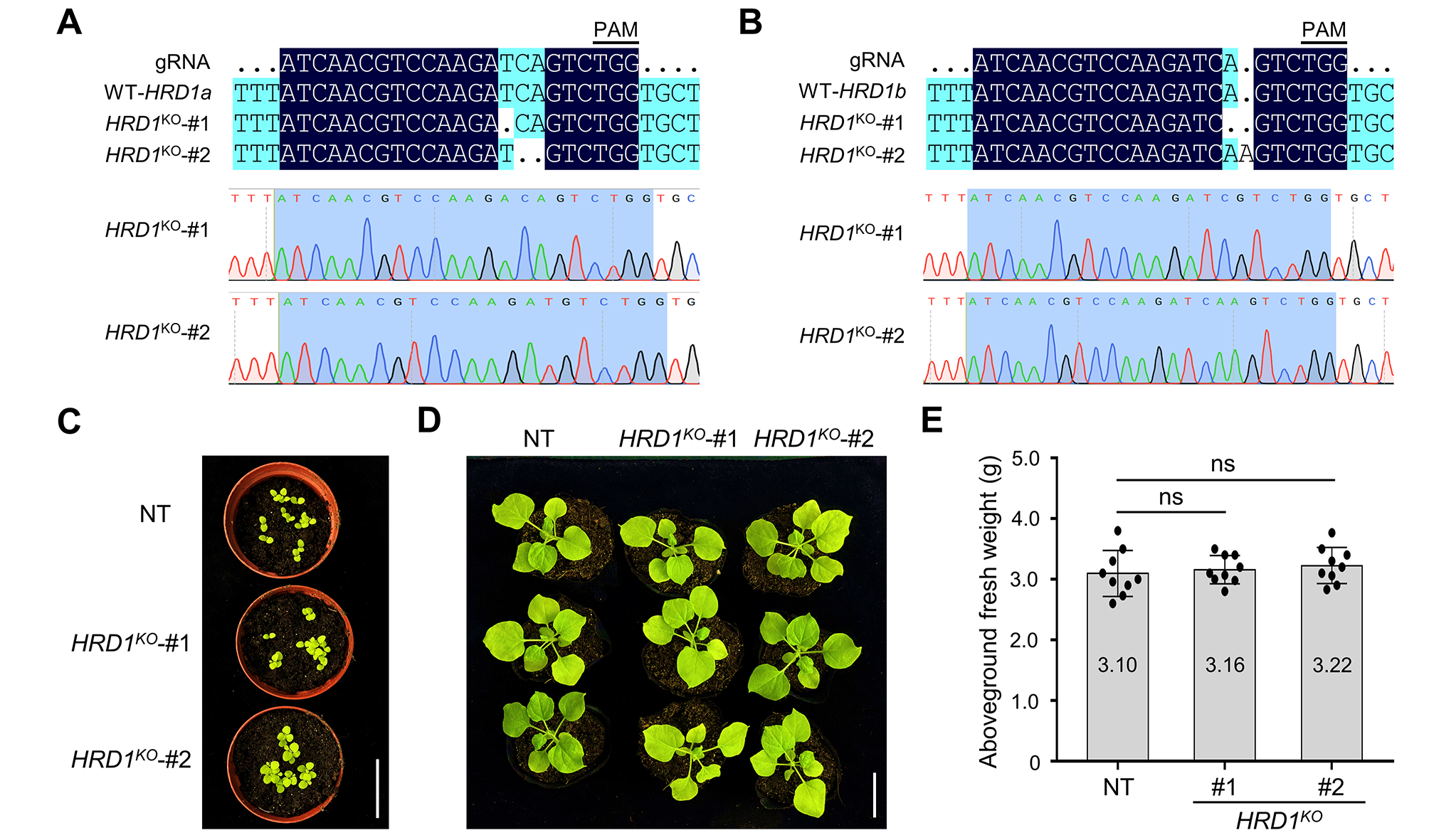

Supplement: S7 Fig — (C) Representative images of 2-week-old NT and two NbHRD1KO lines (#1, #2). Scale bars, 2.5 cm. (D) Representative images of 4-week-old NT and two NbHRD1KO lines (#1, #2). Scale bars, 5 cm. (E) Above ground fresh weight of plants for panel (D). Error bar represents ±SD of 9 plants. Ns, not significance. (Student’s t‐test). (TIF) [file ppat.1013840.s009.tif]
